# Profiling Novel Quinuclidine-Based Derivatives as Potential Anticholinesterase Drugs: Enzyme Inhibition and Effects on Cell Viability

**DOI:** 10.3390/ijms25010155

**Published:** 2023-12-21

**Authors:** Suzana Žunec, Donna Vadlja, Alma Ramić, Antonio Zandona, Nikola Maraković, Iva Brekalo, Ines Primožič, Maja Katalinić

**Affiliations:** 1 Division of Toxicology, Institute for Medical Research and Occupational Health, Ksaverska c. 2, 10000 Zagreb, Croatia; suzana@imi.hr (S.Ž.); azandona@imi.hr (A.Z.); nmarakovic@imi.hr (N.M.); 2 Armed Forces of the Republic of Croatia, Trg Kralja Petra Krešimira IV br. 1, 10000 Zagreb, Croatia; donna.vadlja@gmail.com; 3 Faculty of Science, University of Zagreb, Horvatovac 102a, 10000 Zagreb, Croatia; aramic@chem.pmf.hr (A.R.); i.brekaloiva@gmail.com (I.B.); ines.primozic@pmf.hr (I.P.)

**Keywords:** alcohols, oximes, AChE, BChE, molecular docking, cytotoxicity, pharmacophore

## Abstract

The cholinergic system, relying on the neurotransmitter acetylcholine (ACh), plays a significant role in muscle contraction, cognition, and autonomic nervous system regulation. The enzymes acetylcholinesterase, AChE, and butyrylcholinesterase, BChE, responsible for hydrolyzing ACh, can fine-tune the cholinergic system’s activity and are, therefore, excellent pharmacological targets to address a range of medical conditions. We designed, synthesized, and profiled 14 *N*-alkyl quaternary quinuclidines as inhibitors of human AChE and BChE and analyzed their impact on cell viability to assess their safety in the context of application as potential therapeutics. Our results showed that all of the 14 tested quinuclidines inhibited both AChE and BChE in the micromolar range (*K*_i_ = 0.26 − 156.2 μM). The highest inhibition potency was observed for two bisquaternary derivatives, **7** (1,1′-(decano)bis(3-hydroxyquinuclidinium bromide)) and **14** (1,1′-(decano)bis(3-hydroxyiminoquinuclidinium bromide)). The cytotoxic effect within 7–200 μM was observed only for monoquaternary quinuclidine derivatives, especially those with the C12–C16 alkyl chain. Further analysis revealed a time-independent mechanism of action, significant LDH release, and a decrease in the cells’ mitochondrial membrane potential. Taking all results into consideration, we can confirm that a quinuclidine core presents a good scaffold for cholinesterase binding and that two bisquaternary quinuclidine derivatives could be considered as candidates worth further investigations as drugs acting in the cholinergic system. On the other hand, specific cell-related effects probably triggered by the free long alkyl chain in monoquaternary quinuclidine derivatives should not be neglected in future *N*-alkyl quaternary quinuclidine derivative structure refinements. Such an effect and their potential to interact with other specific targets, as indicated by a pharmacophore model, open up a new perspective for future investigations of these compounds’ scaffold in the treatment of specific conditions and diseases other than cholinergic system-linked disorders.

## 1. Introduction

The cholinergic system is a crucial part of the nervous system that relies on the neurotransmitter acetylcholine (ACh) [1]. Since it plays a significant role in various bodily functions, including muscle contraction, cognition, and autonomic nervous system regulation, the components of the cholinergic system, primarily the enzymes cholinesterases, are excellent pharmacological targets. There are two structurally and functionally related cholinesterases (acetylcholinesterase, EC 3.1.1.7, AChE, and butyrylcholinesterase, EC 3.1.1.8, BChE) responsible for hydrolyzing ACh [2]. AChE is an essential enzyme in cholinergic neurotransmission, and its reversible inhibition has a therapeutic relevance in several neurodegenerative disorders like Alzheimer’s disease (AD), myasthenia gravis, and glaucoma, among others [3]. In contrast, irreversible inhibition of AChE leads to severe toxic effects due to the accumulated ACh [3]. To combat this, AChE-activity-reactivating drugs have been developed to remove the offending AChE inhibitor and restore ACh levels to normal [4]. On the other hand, BChE’s physiological role is still unclear [5,6]. A complementary role for AChE and BChE in the neuromuscular junction has been suggested as AChE is involved in the initial rapid response, but is susceptible to inhibition at high ACh levels, while BChE, which is not inhibited, may operate behind AChE, cleaning up the residual excess neurotransmitter ACh [7]. These data established the basis for the cholinesterase-acting drugs as the most prevalent therapeutics primarily for the symptomatic treatment of AD patients but also for other disorders with cholinergic neurotransmission-linked pathology [8]. It should be noted that in addition to the correlation of senile dementia with ACh deficiency in the brain, ACh was found to be a fine-tuner of inflammation, which raises the possibility that cholinesterase inhibitors may also operate as anti-inflammatory agents [9]. On the other hand, discovery of a new drug and placing it into medical practice are challenging processes requiring overcoming numerous obstacles. In this regard, it is very important that the objective assessment of the effectiveness and safety of a potential drug is clear already in the early phases of drug design and discovery. Here, employing diverse cell-based in vitro assays can help in identification of possible adverse effects or even in the identification of novel drug targets, extending the proposed therapeutic approach. Such an aspect is of a special interest in the ongoing research in the fields of pharmacology and neurobiology directed to develop drugs that can fine-tune cholinergic activity to address a range of medical conditions [8,10].

In the creation of new drug molecules, one of the most widely utilized methods is the modification of the structure of known active compounds or the most important mediators of desired biochemical processes [11]. The group of synthetic quinuclidine derivatives is a very good example of compounds prepared in that way [12]. Due to its highly symmetrical structure, the heterocyclic system of quinuclidine (1-azabicyclo[2.2.2]-octane) is extremely chemically stable and is present as a structural unit of many natural and synthetic physiologically active substances [13]. For instance, four major *Cinchona* alkaloids (quinine, quinidine, cinchonine, and cinchonidine), known for antimalarial activity, contain a quinuclidine moiety [14]. Synthetic quinuclidine-based derivatives have shown a wide spectrum of biological and pharmacological activities such as anticholinergic, antihistamine, antiparasitic, antioxidative, and antitumor activity [15,16,17,18]. Furthermore, several derivatives have shown anticholinesterase activity [19,20], while the ones with the oxime functional group and the alkyl chain in the structure have been classified as a nonconventional class of surfactants [21] and promising antimicrobial agents [21,22].

Taking this together, we focused our study on profiling 14 *N*-alkyl quaternary quinuclidine derivatives as potential anticholinesterase drug scaffolds. Compounds were divided into two groups differing in the functional group at the quinuclidine ring, where one series included a hydroxyl group and the other an oxime group. Considering the potential of molecules with such a combination of functionalities for a wider-scale implementation, we profiled all compounds as reversible inhibitors of human AChE and BChE and analyzed their influence on the cell viability to obtain an insight into the potential of *N*-alkyl quaternary quinuclidines to be developed as new drugs.

## 2. Results

### 2.1. Synthesis of Compounds

**Figure 1:**
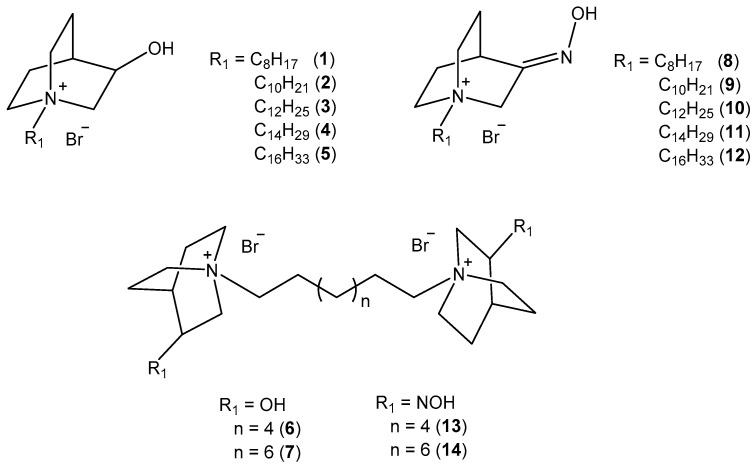
Chemical structures of designed and synthesized *N*-alkyl quaternary 3-substituted quinuclidines (**1**–**14**) tested in this study.

We have designed and synthesized quaternized 3-substituted quinuclidines with variations in N-alkyl chain length and incorporation of an alcohol or oxime headgroup at position 3 of the quinuclidine ring (compounds **1**–**14**; Figure 1). Six (**6**–**9**, **13**, and **14**) out of fourteen compounds have not been described in the literature so far.

Quinuclidine-3-oxime (QNOH) was prepared by the reaction of quinuclidine-3-one and hydroxylamine hydrochloride in basic solution as described previously [23], while quinuclidine-3-ol (QOH) is a commercially available chemical. N-alkyl monoquaternary derivatives **1**–**12** were prepared by reaction of QOH or QNOH and appropriate alkyl bromide in dry acetone. Bisquaternary derivatives (**6**, **7**, **13**, and **14**) were prepared by the reaction of appropriate 3-substituted quinuclidine and 1,8-dibromooctane or 1,10-dibromodecane in dry methanol. All synthesized compounds were obtained as white solids in satisfactory yields. Their structures were confirmed by the standard analytical methods (1H and 13C 1D and 2D NMR).

### 2.2. Reversible Inhibition of AChE and BChE with N-Alkyl Quaternary Quinuclidines

All of the tested compounds reversibly inhibited both AChE and BChE activity in the micromolar range, and the determined inhibition constants *K*_i_ are summarized in Table 1. Analysis of the impact of the functional group has revealed following: (i) mono- and bisquaternary oxime quinuclidines with C8 and C10 alkyl chains were more potent reversible inhibitors of both ChEs compared to their alcohol analogs; (ii) oxime and alcohol quinuclidines with a C12 chain had equal potential for reversible AChE inhibition, while the oxime analog was 1.5-fold better as a reversible inhibitor of BChE; (iii) quinuclidine alcohols with longer alkyl chains, specifically C14 and C16, were more potent reversible inhibitors of both ChEs compared to their oxime analogs. Furthermore, according to the ratio of *K*_i_ (AChE) over *K*_i_ (BChE), selectivity for binding was also observed in terms of alkyl chain length. While AChE favored compounds with alkyl side chains of C14 and C16, BChE displayed a different selectivity pattern, showing a preference for compounds with shorter alkyl chains of C8 and C10. On the other hand, the most potent inhibitors were bisquaternary quinuclidines, alcohol (**7**) and oxime (**14**), both with a C10 alkyl linker (*K*_i_ = 0.2 − 1.6 µM). Both compounds **7** and **14** were more selective for inhibiting AChE over BChE.

### 2.3. Molecular Modeling of AChE and BChE in Complex with N-Alkyl Quaternary Quinuclidines

**Figure 2:**
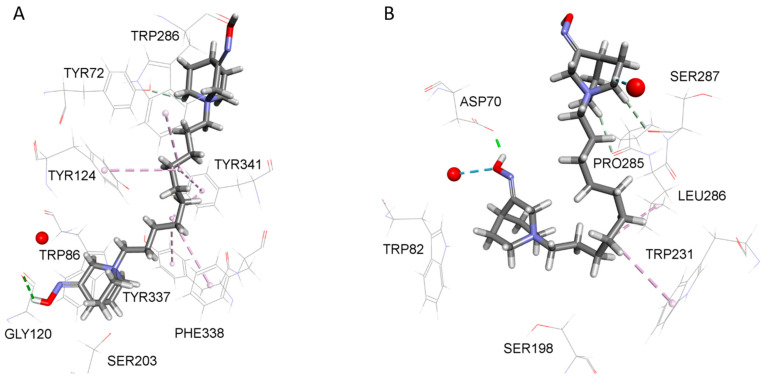
Close-up of AChE (**A**) and BChE (**B**) active site from model complexes with compound **14** selected based on the highest potency for reversible inhibition of both enzymes. Dashed lines represent different types of non-bonding interactions (magenta—electrostatic, green and blue—hydrogen bonds). Used for the study: crystal structures of free AChE (PDB ID: 4EY4) [25] and BChE (PDB ID: 1P0I) [26].

The positioning and interactions of *N*-alkyl quaternary quinuclidines within the cholinesterases’ active site were evaluated by molecular docking analysis. A list of interactions between tested quinuclidines and AChE is given in Tables S1–S14 and, for BChE, in Tables S15–S28. Figure 2 shows a close-up representative view of the AChE and BChE active site from model complexes with compound **14**.

As the results revealed, the narrow active site gorge of AChE (200 Å^3^ smaller in volume compared to BChE) leaves less available space for quinuclidine ligands to freely rotate inside the active site and adopt a bent conformation with long hydrocarbon chains. Indeed, the representative poses of model complexes between *N*-alkyl quaternary quinuclidine oximes and AChE predict that only compound **8** binds in an orientation characterized by the oxime-bearing quinuclidine ring placed between the choline-binding region and catalytic triad, while the attached hydrocarbon chain adopts a downward curled structure directing its terminal methyl group to interact with distinct Trp236 residue. In the case of AChE, all oximes with a hydrocarbon chain longer than 8 methyl units are predicted to bind in an orientation with elongated conformation of the hydrocarbon chain and directed at the active site gorge opening. As expected, in the case of model complexes between *N*-alkyl quaternary quinuclidine alcohols and AChE, all compounds are predicted to bind in an orientation with a more elongated hydrocarbon chain/linker, though, in the case of alcohol **1**, the hydrocarbon chain to a certain degree displayed a twisted structure.

The representative poses of model complexes between *N*-alkyl quaternary quinuclidine oximes and BChE predict that oximes occupy the active site in an orientation characterized by the oxime-bearing quinuclidine ring being placed in the vicinity of the choline-binding region and catalytic triad, while the long hydrocarbon chain adopts a downward curled structure to an extent that enables its terminal methyl group to interact with distinct Trp231 residue. However, the ability of a long hydrocarbon chain to orient inside the active site in such a manner is dependent both upon the length of the hydrocarbon chain and the spatial requirements set by the BChE active site three-dimensional proportions. A relatively large volume of BChE active site (approx. 500 Å^3^) [24] allows for compounds with a hydrocarbon chain of up to 12 methyl units (C8 to C12) to adopt such an orientation, while compounds with longer hydrocarbon chains (C14 and C16) are predicted to either bind with the oxime-bearing quinuclidine ring placed in the vicinity of the gorge opening and the corresponding hydrocarbon chains protruding to the bottom of the active site, adopting an L-shaped structure bent in the proximity of the distinct Trp82 residue with the remaining part of the hydrocarbon chain directed to the center of active site **11**, or bind in an orientation with elongated conformation of hydrocarbon chain and directed at the gorge opening while the quinuclidine ring remains in the lower part of active site **12**. Interestingly, bis oximes **13** and **14**, which can tentatively be seen as having 12 and 14 methyl units attached to the nitrogen atom of the quinuclidine ring occupying the lower part of the active site (if one continues counting across the bridge of the second quinuclidine ring located in the upper side of the active site and includes the second nitrogen atom in the count), seem to adhere to this finding in a similar way. Indeed, **13**, which can correspond to compound **10** when applying the above-mentioned analogy in the number of apparent methyl units, adopts a more bent conformation, with the second ring leaning to the wall of the active site gorge and the oxime group pointing at Asp70, with which it engages in interaction. On the other hand, **14**, corresponding to **11** in terms of the apparent methyl units, is predicted to be oriented in a more elongated conformation with its second ring directed at the gorge opening (Figure 2A).

Unsurprisingly, in the case of model complexes between *N*-alkyl quaternary quinuclidine alcohols and BChE, even compounds with hydrocarbon chains which are 14 and 16 methyl units long are predicted to accommodate inside the BChE active site with the alcohol-bearing quinuclidine ring at the bottom of the active site and bent conformation of the hydrocarbon chain. In both instances, the alcohol-bearing quinuclidine ring is located deeper in the active site gorge in comparison with their analog compounds with oxime-bearing quinuclidine ring, which could explain differences in the binding between these compounds per se. At the same time, both bis alcohols **6** and **7** are predicted to bind in an orientation with a hydrocarbon linker between two rings in a more elongated conformation. In the case of **6**, this is facilitated by the ring located in the lower part of the active site gorge being shifted closer to the bottom center.

### 2.4. Effects of N-Alkyl Quaternary Quinuclidines on Cells’ Viability

#### 2.4.1. Cytotoxic Effect of Quinuclidine Derivatives

Cytotoxicity of tested quinuclidines was evaluated on neuron-like SH-SY5Y and hepatocyte HepG2 cell lines in order to investigate whether they trigger unwanted effects. The concentration range tested in the experiments was selected to correspond to the physiologically relevant concentrations for cholinesterase-based ligand usage [27,28,29], as well as the concentration range used here in the in vitro kinetic experiments. As the results indicate, after 24 h of cell exposure, six compounds, **3**, **4**, **5**, **10**, **11**, and **12**, showed cytotoxic effects on both cell lines up to 200 µM (Table 2). Here, the cytotoxicity increased with the length of the chain on the quinuclidine core from C12 to C16. Furthermore, SH-SY5Y cells were more susceptible to alcohol and HepG2 cells to the oxime analogs. Additional analysis following 1 and 4 h of exposure revealed time-independent cytotoxicity profiles for majority of the toxic derivatives (Figures S13 and S14).

#### 2.4.2. Damage of the Cell Membrane Integrity

**Figure 3:**
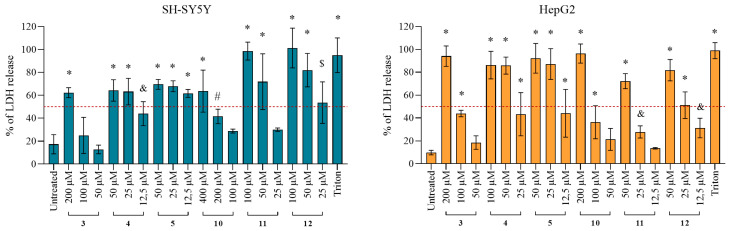
Levels of LDH release after 4 h exposure to selected quinuclidine compounds **3**–**5**, **10**, **11**, and **12**. Cells were exposed to the selected compounds in concentrations of 12.5–200 µM. Statistical significance: ordinary one-way ANOVA Dunnett’s multiple comparisons test to untreated control (& *p* ≤ 0.05; # *p* ≤ 0.01; $ *p* ≤ 0.001; * *p* ≤ 0.0001). Triton X-100 (0.18%) was used as a positive control. The red dashed line represents 50% of LDH released from cells. Results are presented as a percentage of LDH release compared to the determined maximal LDH release with Triton.

According to the observed cytotoxic effect for alcohol and oxime analogs **3**, **4**, **5**, **10**, **11**, and **12**, with C12, C14, and C16 side chains, we further tested the cell membrane integrity after exposure to these compounds following the release of the lactate dehydrogenase (LDH) enzyme from the cytosol to the cultivation medium. Obtained results are presented in Figure 3 and show that all of the analogs caused a significant release of LDH (Figure 3), which is an indication of compromised cell membrane integrity. The strongest effect was observed for alcohols **3**, **4**, and **5** on HepG2 and oximes **10**, **11**, and **12** on SH-SY5Y cells.

#### 2.4.3. Changes in the Mitochondrial Membrane Potential

**Figure 4:**
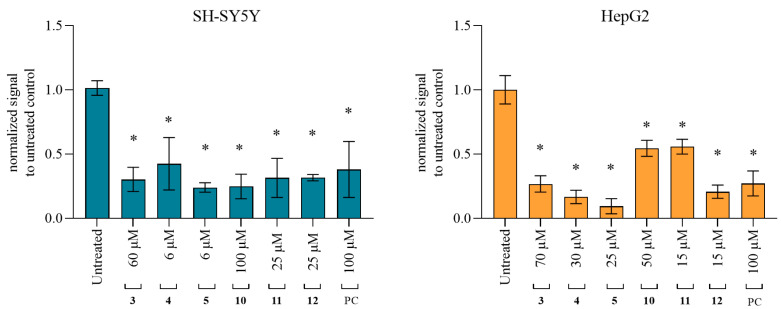
Mitochondrial membrane potential (ΔΨ_m_) determined by TMRE assay after 4 h exposure to selected quinuclidine compounds **3**–**5**, **10**, **11**, and **12**. Cells were exposed to LOAEL concentrations of the selected compounds. Statistical significance: ordinary one-way ANOVA Dunnett’s multiple comparisons test to untreated control (* *p* < 0.0001). CCCP (100 µM) was used as a positive control (PC), inducing a decrease in ΔΨm compared to untreated controls. Results are presented as relative fluorescence units (RFU) to control.

Since cytotoxicity determination was based on the mitochondrial succinate dehydrogenase activity assay, we wanted to further see whether quinuclidines exhibit mitotoxicity. The results presented in Figure 4 indicate that all six tested cytotoxic compounds affected mitochondrial membrane potential (ΔΨ_m_), indicating interference with a proper mitochondrial function and energy metabolism. The strongest effect was observed for quinuclidine alcohol **5** on HepG2.

#### 2.4.4. Activation of the Caspases as the Specific Apoptotic Markers

**Figure 5:**
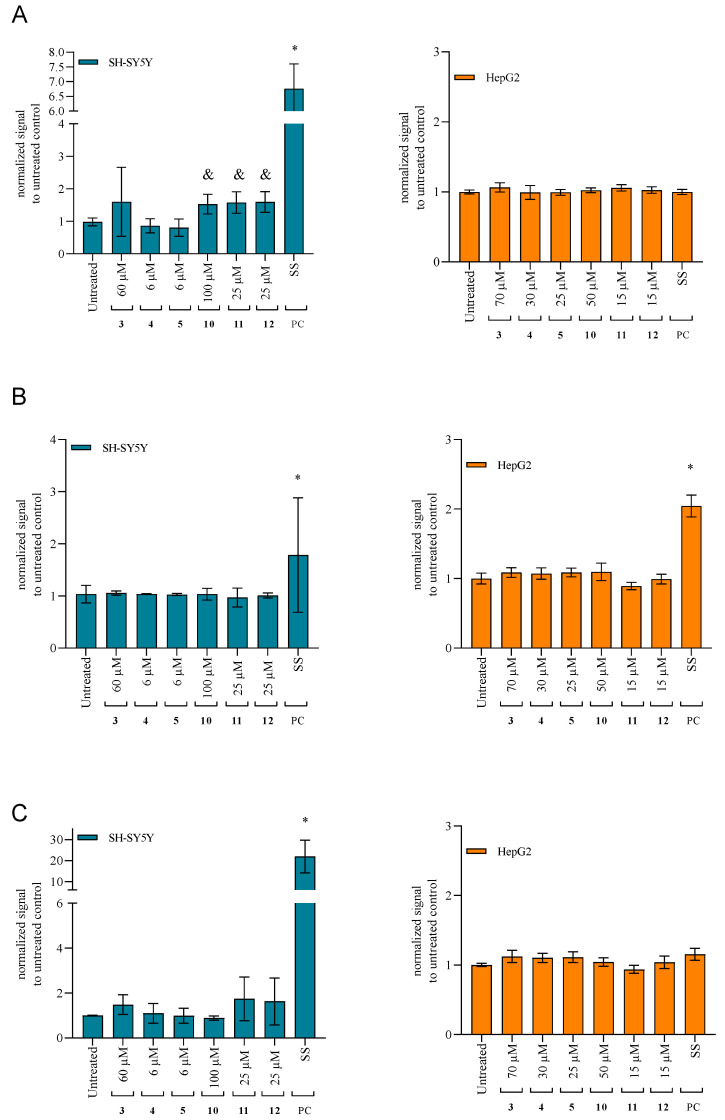
Specific caspase activities after 4 h treatment with the tested quinuclidine compounds **3**–**5**, **10**, **11**, and **12**: (**A**) caspase 8 on SH-SY5Yand HepG2, (**B**) caspase 9 on SH-SY5Y and HepG2, (**C**) caspase 3 on SH-SY5Y and HepG2. Cells were exposed to LOAEL concentrations of the selected compounds. Statistical significance: ordinary one-way ANOVA Dunnett’s multiple comparisons test to untreated control (& *p* ≤ 0.05; * *p* ≤ 0.0001). Staurosporine (SS, 3 µM) was used as a positive control (PC). Results are presented as relative fluorescence units (RFU) to control untreated cells.

To further evaluate the obtained results from the performed cell-based assays, we analyzed whether cytotoxic quinuclidine compounds trigger apoptosis by following specific caspases as apoptotic markers. For this, we selected to follow initiator caspase 8 (activated by the membrane receptor-specific signal), caspase 9 (activated by the mitochondria-specific signal), and executor caspase 3 (activated downstream by caspases 8 and 9). From the results shown in Figure 5, it is evident that, in the tested time-frame, only significant activation of caspase 8 was observed and only by three cytotoxic oxime analogs, **10**, **11**, and **12**, in SH-SY5Y (Figure 5A).

## 3. Discussion

Developing new drugs acting on the cholinergic system to fight neurodegenerative disorders such as Alzheimer’s disease and myasthenia gravis occupies a central role in numerous ongoing pieces of research. Here, the inhibition of the neurotransmitter acetylcholine hydrolyzing enzymes AChE and BChE has emerged as a strategy to pursue [8]. However, though the two share the same catalytic mechanism of hydrolysis of choline esters, finding an efficient inhibitor of each of the enzymes can be challenging due to the intrinsic differences related to the specificity for substrates, reversible ligands, and irreversible inhibitors [5,32,33].

We have tested a series of 14 *N*-alkyl quaternary quinuclidine derivatives, of which six (6) were not previously described and/or mentioned in the literature (cf. Figure 1). We aimed to investigate how different chemical groups (oximes and alcohols), or variation in the alkyl chain length, within a molecule of quaternized quinuclidine core can influence the compound’s biological activity. Namely, we performed detailed in vitro analysis of *N*-alkyl quaternary quinuclidines’ anticholinesterase activity and potential toxic effects on selected cells to unriddle their potential for therapeutic applications and inform decisions regarding their future use.

According to the results, all 14 tested *N*-alkyl quaternary quinuclidines had a potency to inhibit both AChE and BChE in the micromolar range, placing them in line with currently investigated promising cholinesterase inhibitors of another structural cores [34,35]. In our case, it seems that the greatest influence on the inhibition property was observed when increasing the alkyl chain length from C8 to C16, whereas C14 was determined as the most optimal one to interact with the cholinesterase active site. The influence of the oxime over the alcohol functional group was the most pronounced for compounds with the smallest alkyl chain (C8), as well as for their bis conjugates. The highest potency for inhibition of both AChE and BChE was observed for bisquaternary quinuclidine compounds **7** and **14** (C10 alkyl linker), with an inhibition constant (*K*_i_) in the nanomolar range ensuring their further consideration as possible drug candidates and pointing once again to a quinuclidine core as a promising starting point in the cholinesterase inhibitors’ design [30,36]. However, if we compare our strongest inhibitor, **14**, to donepezil as the medically approved acetylcholinesterase reversible inhibitor for the treatment of neurodegenerative disorders [37,38,39,40], it is still around 60 times less efficient. Namely, the *K*_i_ of donepezil determined in previous studies was 0,0043 μM [39]. Interestingly, when looking at the selectivity, the highest ratio of five-fold AChE/BChE, or vice versa, was also observed for C10-chain compounds. Although this selectivity is rather modest, it could be indicative for future structure refinement if creating compounds with a therapeutic advantage in situations where selective inhibition of AChE or BChE is desired.

Though it is challenging to correlate such inhibition results obtained experimentally with the molecular docking analysis, certain structural features and properties of *N*-alkyl quaternary quinuclidines appeared to increase their probability for stronger binding to both enzymes: total number of non-bonding interactions (the full list of non-bonding interactions for a particular compound is listed in Tables S1–S28), the number of electrostatic (attractive) interactions and conventional hydrogen bonds, and the number of non-bonding interactions with residues from the catalytic triad. Additionally, in the case of AChE, the inhibition potency correlated with the number of interactions involving conserved residues along the active site gorge wall that play an important role in substrate trafficking, such as Trp286 from the peripheral active site (PAS) region (important for initial substrate binding) and Tyr124 (one of two residues defining the bottleneck region midway down the active site gorge that undergoes significant distortion to allow the substrate to access the catalytic triad at the bottom of the gorge) [41,42]. Also, it seems that AChE favors compounds predicted to bind in elongated conformation, which enables simultaneous binding to both the catalytic site and PAS region rather than the curled or bent structure of a bound compound. However, when strictly adhering to any of these properties alone, one could not explain the experimental trends in inhibition potencies among tested compounds. Thus, the most plausible explanation could be that each of these properties carries a special weight when predicting the inhibition potency of a particular compound, and it is, therefore, the weighted sum of individual contributions from these properties that predicts the inhibition potency rather than any individual property. Moreover, a particular property with a high value can compensate for a lack of another more important property, e.g., a very high total number of non-bonding interactions can compensate for the lack of non-bonding interactions with any of the residue from the catalytic triad. Finally, one should consider limitations of our approach when considering chiral molecules in the case of alcohols. Namely, for this study, only the enantiomer with the highest score was subjected to analysis. It is unclear to what extent an enantiomer with a lower score actually contributes to experimentally determined inhibition potency.

Furthermore, we evaluated the tested *N*-alkyl quaternary quinuclidines’ impact on the cell viability to assess their safety and potential harmful effects in the context of their suitability for specific applications or as potential therapeutics. Such assessment could be essential to understand whether any observed cytotoxicity is linked to the specific structural features of the compound and/or the specific cells exposed. In our case, 6 out of 14 analogs showed a cytotoxic effect in the studied concentration range. Here, we have to emphasize that toxicity was observed only for monoquaternary derivatives but not for four bisquaternary analogs, **6**, **7**, **13**, and **14**, which showed the highest cholinesterase inhibition potency. For those six toxic derivatives (**3**, **4**, **5**, **10**, **11**, **12**), we have analyzed the effects on cells further. Similarly, as for the ChEs inhibition potency, we observed here that the length of the alkyl chain on the quinuclidine core played a significant role in affecting both neuron-like SH-SY5Y and hepatocyte HepG2 cells. Toxicity of the compounds increased with the increase in the alkyl chains (specifically C12, C14, and C16) for both groups, alcohols and oximes. Interestingly, SH-SY5Y cells were more affected by the alcohol than by the oxime analogs. The toxicity result confirms previous studies from other researchers reporting on quaternary ammonium compound derivatives with longer alkyl chains having greater toxic effect toward human tumor and healthy cells [43,44]. Looking at the structure of such toxic compounds, we could link this effect to the aliphatic side chain itself and its probability to mimic a fatty acid and interfere with cell membrane stability [44]. Namely, previous studies have shown that compounds with a hydroxyimino and hydroxyl quniclidinium headgroup and variation in alkyl chain length act as potential surfactants also affecting bacterial cell membrane integrity [21,22]. Our further cytotoxicity analysis after 1 and 4 h of cell exposure revealed time-independent effects in both cell lines for majority of the six toxic compounds. The time-dependent effect was pronounced after treatment with alcohol and oxime analogs with a C12 chain, suggesting a type of regulated cell death like apoptosis as a possible additional mechanism of action [45]. Since the compounds probably act on the cell membranes, we hypothesize that they could activate specific receptors on the outer membrane and trigger apoptosis, initiating a cascade of reactions governed by the caspase enzymes [45]. However, in the studied time and concentration range, we observed activation of the initiator caspase 8 only in SH-SY5Y cells. Additionally, further tests showed that all six cytotoxic quinuclidines (**3**, **4**, **5**, **10**, **11**, **12**) triggered significant LDH release and a decrease in the mitochondrial membrane potential, indicating membrane damage to the cells. This possible membrane rupture is probably the strongest effect of the six tested toxic compounds and defines their overall action on the cell level. This is supported by the study of Dymond et al. [46], which postulated that cytotoxicity of amphiphilic compounds including quinuclidinium derivatives is mediated through a reduction in membrane curvature elastic stress (which is a lipid bilayer property that plays a role in maintaining the functionality of several membrane-associated proteins). On the other hand, it is reasonable to assume that the four bisquaternary quinuclidine compounds **6**, **7**, **13**, and **14**, showing no toxic effect to cells up to 800 μM, would not induce such changes, which makes them candidates worth further investigations as drugs acting in the cholinergic system. As this opens up a new perspective for those four derivatives, especially derivatives **7** and **14**, other monoquaternary quinuclidine derivatives could be investigated in the future beyond the anticholinesterase action. Therefore, we screened the known databases according to the generated pharmacophore model (see Supplementary Materials) to find similar compounds with defined targets which could also be potential targets of our compounds’ action. Based on the ligand mapping onto the pharmacophore model, a significant similarity was found with a reversible carnitine palmitoyltransferase inhibitor with antiketotic and antidiabetic activity, namely, 3-(tetradecylcarbamoylamino)-4-(trimethylazaniumyl)butanoate (see Figure S15).

In addition to the cholinesterase reversible inhibition, the *N*-alkyl quaternary quinuclidine oxime-bearing analogs could be tested as reactivators of irreversibly inhibited or phosphylated cholinesterases as well. Namely, irreversible AChE inhibitors such as toxic organophosphorus compounds, used as pesticides and nerve warfare agents, still present a threat for human health and are still without an adequate treatment [47]. The introduction of an oxime group into an appropriate chemical backbone has produced very effective AChE reactivators, various kinase inhibitors, and compounds with antibacterial, anticancer, anti-inflammatory, anti-arthritis, and anti-stroke activities [48,49]. Overall, these findings highlight the importance of the choice of the functional group and the alkyl chain length in designing ChEs inhibitors based on a quinuclidine scaffold as they can have a significant impact on the positive biological potency of compounds.

## 4. Materials and Methods

### 4.1. Synthesis of N-Alkyl Quaternary Quinuclidines

#### 4.1.1. Chemicals

**Figure 6:**
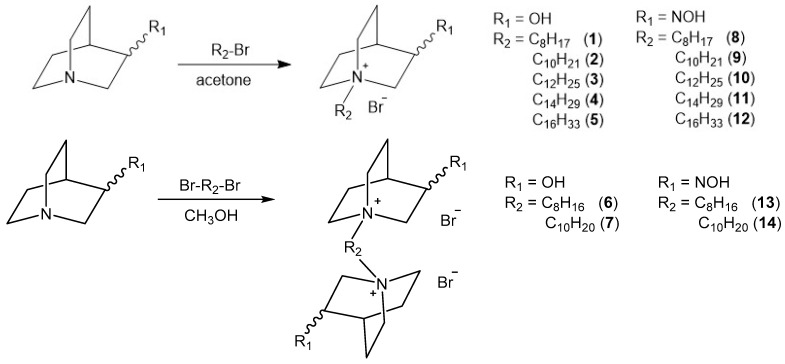
Schematic representation of synthetic routes for monoquaternary and bisquaternary compounds **1**–**14**.

Chemicals, reagents, and solvents used for the preparation of compounds were purchased from Sigma-Aldrich (St. Louis, MO, USA). The reactions were monitored by thin-layer chromatography plates coated with silica gel (Sigma-Aldrich, St. Louis, MO, USA). TLC plates were visualized by UV irradiation (254 nm) or by iodine fumes. One-dimensional and two-dimensional ^1^H and ^13^C NMR spectra (Figures S1–S12) were recorded on a Bruker Avance III HD 400 MHz/54 mm Ascend spectrometer (Bruker Optics Inc., Billerica, MA, USA). Chemical shifts are given in ppm downfield from tetramethylsilane (TMS) as an internal standard and coupling constants (J) in Hz. Splitting patterns are labeled as s (singlet), quin (quintet), or m (multiplet). For compounds **6**, **7**, **13**, and **14**, hydrogen and carbon atoms of the second quinuclidine ring are marked with prime. Alkyl hydrogen and carbon atoms are marked with a double prime (Figure 6). Melting points were determined on a Melting Point B-540 apparatus (Büchi, Essen, Germany) and are uncorrected. HRMS analyses were carried out on Q Exactive™ Plus Hybrid Quadrupole-Orbitrap™ Mass Spectrometer. Synthesis of compounds **1**–**5** [22] and **10**–**12** [21,30] was published previously (Figure 6).

#### 4.1.2. General Procedure for Preparation of QOH and QNOH Monoquaternary Derivatives

QOH or QNOH (1 mmol) was dissolved in dry acetone, and appropriate alkyl bromide (1.5 mmol) was added slowly. The mixture was heated for 24 h at 50 °C. After cooling to room temperature, white solid product was filtered off and washed several times with dry diethyl ether.

#### 4.1.3. General Procedure for Preparation of QOH and QNOH Bisquaternary Derivatives

QOH or QNOH (1 mmol) was dissolved in dry methanol, and appropriate alkyl dibromide (0.5 mmol) was added slowly. The mixture was heated for 24 h at 50 °C. After cooling to room temperature, the solvent was evaporated. Solid product was recrystallized from diethyl ether.

1,1′-(octano)bis(3-hydroxyquinuclidinium bromide) (**6**): Procedure 4.1.2. White solid, yield 92%; mp 235–236 °C; ^1^H NMR (400 MHz, MeOD-*d*_4_) δ/ppm: 1.34–1.52 (m, 8 H, H3″-H6″); 1.69–2.20 (m, 12 H, H5, H5′, H7, H7′, H2″, H7″); 2.28–2.37 (m, 2 H, H4, H4′); 3.08–3.46 (m, 16 H, H2, H2′, H6, H6′, H8, H8′, H1″, H8″); 3.65–3.76 (m, 2 H, H3, H3′); 4.17–4.25 (m, 2 H, OH, OH’). ^13^C NMR (101 MHz, MeOD-d4) δ/ppm: 18.84 (C5, C5′); 22.43 (C7, C7′); 22.97, 27.29, 29.76 (C2″–C7″); 28.06 (C4, C4′); 54.75, 55.97, 64.53, 65.37 (C2, C2′, C6, C6′, C8, C8′, C1″, C8″); 65.46 (C3, C3′). HRMS (electrospray ionization (ESI)) *m*/*z* calcd for C_22_H_42_N_2_O_2_^2+^ = 183.1618, found 183.1617.

1,1’-(decano)bis(3-hydroxyquinuclidinium bromide) (**7**): Procedure 4.1.2. White solid, yield 81%; mp 240–241 °C; ^1^H NMR (400 MHz, MeOD-*d*_4_) δ/ppm: 1.32–1.42 (m, 12 H, H3″–H8″); 1.69–2.20 (m, 12 H, H5, H5′, H7, H7′, H2″, H7″); 2.27–2.37 (m, 2 H, H4, H4′); 3.11–3.48 (m, 20 H, H2, H2′, H6, H6′, H8, H8′, H1″, H8″); 3.66–3.75 (m, 2 H, H3, H3′); 4.18–4.25 (m, 2 H, OH, OH’). ^13^C NMR (101 MHz, MeOD-d4) δ/ppm: 18.84 (C5, C5′); 22.43 (C7, C7′); 23.05, 27.48, 30.05, 30.28 (C2″–C9″); 28.05 (C4, C4′); 54.73, 55.94, 64.50, 65.41 (C2, C2′, C6, C6′, C8, C8′, C1″, C10″); 65.45 (C3, C3′). HRMS (electrospray ionization (ESI)) *m*/*z* calcd for C_24_H_46_N_2_O_2_^2+^ = 197.1774, found 197.1773.

1-octyl-3-hydroxyiminoquinuclidinium bromide (**8**): Procedure 4.1.3. White solid, yield 72%, mp 197–198 °C; ^1^H NMR (400 MHz, MeOD-*d*_4_) δ/ppm: 0.90–0.97 (m, 3 H, H8″); 1.32–1.47 (m, 10 H, H3″–H7″); 1.77–1.87 (m, 2 H, H2″); 2.06–2.18 (m, 2 H, H5); 2.22–2.33 (m, 2 H, H7); 2.91 (quin, *J* = 3.2 Hz, 1 H, H4); 3.35–3.40 (m, 2 H, H1″); 3.50–3.60 (m, 2 H, H8); 3.62–3.72 (m, 2 H, H6); 4.38 (s, 2 H, H2). ^13^C NMR (101 MHz, MeOD-*d*_4_) δ/ppm: 14.55 (C8″); 23.39 (C7″); 23.81 (C6″); 24.74 (C4″, C5″); 27.67 (C7); 28.45 (C4); 30.30 (C3″); 30.35 (C2″); 33.02 (C5); 56.50 (C6, C8); 58.73 (C1″); 65.99 (C2); 152.18 (C3). HRMS (electrospray ionization (ESI)) *m*/*z* calcd for C_15_H_29_N_2_O^+^ = 253.2274, found 253.2271.

1-decyl-3-hydroxyiminoquinuclidinium bromide (**9**): Procedure 4.1.3. White solid, yield 69%; mp 200–201 °C; ^1^H NMR (400 MHz, MeOD-*d*_4_) δ/ppm: 0.88–0.96 (m, 3 H, H10’); 1.29–1.45 (m, 14 H, H3″–H9″); 1.77–1.87 (m, 2 H, H2″); 2.06–2.18 (m, 2 H7); 2.20–2.33 (m, 2 H, H5); 2.91 (quin, *J* = 3.1 Hz, 1 H, H4); 3.35–3.41 (m, 2 H, H1″); 3.55 (m, 2 H, H8); 3.62–3.72 (m, 2 H, H6); 4.39 (s, 2 H, H2). ^13^C NMR (101 MHz, MeOD-*d*_4_) δ/ppm: 14.57 (C10″); 23.40 (C8″, C9″); 23.87 (C7″); 24.74 (C6″); 27.67 (C7); 28.46 (C4); 30.34 (C5″); 30.54 (C4″); 30.68 (C3″); 30.73 (C2″); 33.18 (C5); 56.50 (C6, C8); 58.73 (C1″); 65.98 (C2); 152.20 (C3). HRMS (electrospray ionization (ESI)) *m*/*z* calcd for C_17_H_33_N_2_O^+^ = 281.2587, found 281.2585.

1,1’-(octano)bis(3-hydroxyiminoquinuclidinium bromide): (**13**) Procedure 4.1.3. White solid, yield 63%; mp 200–201 °C; ^1^H NMR (400 MHz, DMSO-*d*_6_) δ/ppm: 1.26–1.36 (m, 8 H, H3’–H6’); 1.67–1.76 (m, 4 H, H2″, H7″); 1.88–2.17 (m, 8 H, H5, H5′, H7, H7′); 2.76–2.84 (m, 2 H, H4, H4′); 3.34–3.66 (m, 12 H, H1″, H8″, H6, H6′, H8, H8′); 4.34 (s, 4 H, H2, H2′); 11.13 (s, 2 H, NOH, NOH’). ^13^C NMR (101 MHz, DMSO-d6) δ/ppm: 21.36, 23.04, 25.75, 28.14 (C5, C5′, C7, C7′, C2″–C7″); 26.50 (C4, C4′); 54.06, 57.06, 63.16 (C2, C2′, C6, C6′, C8, C8′, C1″, C8″); 151.23 (C3, C3′). HRMS (electrospray ionization (ESI)) *m*/*z* calcd for C_22_H_40_N_4_O_2_^2+^ = 196.1570, found 196.1568.

1,1’-(decano)bis(3-hydroxyiminoquinuclidinium bromide): (**14**) Procedure 4.1.3. White solid, yield 69%; mp 212–213 °C; ^1^H NMR (400 MHz, DMSO-*d*_6_) δ/ppm: 1.27–1.33 (m, 12 H, H3″-H8″); 1.66–1.73 (m, 4 H, H2″, H7″); 1.89–2.16 (m, 8 H, H5, H5′, H7, H7′); 2.78–2.82 (m, 2 H, H4, H4′); 3.33–3.65 (m, 12 H, H1″, H10″, H6, H6′, H8, H8′); 4.30–4.39 (m, 4 H, H2, H2′); 11.13 (s, 2 H, NOH, NOH’). ^13^C NMR (101 MHz, DMSO-*d*_6_) δ/ppm: 21.40, 23.06, 25.88, 28.42, 28.64 (C5, C5′, C7, C7′, C2″–C9″); 26.50 (C4, C4′); 54.07 57.06, 63.21 (C2, C2′, C6, C6′, C8, C8′, C1″, C10″); 151.23 (C3, C3′). HRMS (electrospray ionization (ESI)) *m*/*z* calcd for C_24_H_44_N_4_O_2_^2+^ = 210.1727, found 210.1724.

### 4.2. AChE and BChE Reversible Inhibition

#### 4.2.1. Chemicals and Enzymes

Cholinesterase substrate acetylthiocholine (ATCh) and the thiol reagent 5,5′-dithiobis(2-nitrobenzoic acid) (DTNB) for the enzyme activity measurement were purchased from Sigma-Aldrich (St. Louis, MO, USA). The sources of human AChE and BChE were native erythrocytes and plasma, respectively, obtained from the blood of a healthy male volunteer aged 30 and in accordance with the approval of the Ethics Committee of the Institute for Medical Research and Occupational Health (approval number: 100-21/23-6). Erythrocytes and plasma were isolated from the whole blood and prepared according to a previously published procedure [50,51]. Namely, venous blood was collected into a heparinized tube and centrifuged (at 4 °C, 20 min on 2500 rpm, Eppendorf centrifuge 5702R) to separate plasma from the erythrocytes. Plasma was removed and used as a source of BChE, while erythrocytes were washed twice with the 0.1 M sodium phosphate buffer, pH 7.4, in a volume corresponding to the initial volume of the whole blood. Both plasma and erythrocytes were frozen at −20 °C until use. Quinuclidine compounds were dissolved in dH_2_O as 10 or 100 mM; exceptionally, 3-hydroxyimino compounds with longer chains (C12−C16) were dissolved in DMSO as 100 mM solutions. Stock solutions were kept at 4 °C until use. Further dilutions were made in distilled water. The final DMSO concentration in the experiments did not exceed 0.01% and did not affect the enzyme activity measurements.

#### 4.2.2. The Enzyme Activity Measurement and Determination of the Reversible Inhibition by Quinuclidine Compounds

The reversible inhibition experiments followed a previously published procedure [49,52]. Namely, the enzyme activity was measured in the presence of a wide range of quinuclidine compound concentrations and in the substrate range from 0.1 to 0.5 mM to confirm the type of compound binding. The activity was assayed following the Ellman’s procedure [53,54], with the modification in the wavelength used for detection of the TNB anion in the case of AChE activity measurement due to the interference of the hemoglobin, and according to Worek et al. [54]. The assay was performed in 96-well plates on an Infinite M200PRO plate reader (Tecan Austria GmbH, Salzburg, Austria) at 25 °C. Each concentration was tested in triplicate or quadruplicate on each plate. The inhibition mixture contained 0.1 M sodium phosphate buffer, pH 7.4, enzyme (AChE or BChE), quinuclidine compound (0.2–400 μM final), DTNB (0.3 mM final), and ATCh (from 0.1 to 0.5 mM final) to start the reaction. The increase in absorbance was read at 436 nm for AChE and at 412 nm for BChE. The measured activity in the presence of compounds was corrected for the non-enzymatic hydrolysis of substrate ATCh, if detected [55]. The reversible enzyme–quinuclidine complex dissociation constants (the inhibition constants) *K*_i_ were evaluated from the effect of the substrate concentration on the degree of inhibition according to the Hunter–Downs equation as described previously [56], taking into account only quinuclidine concentrations where 20–80% inhibition of the activity was observed.

### 4.3. In Silico Molecular Docking Studies

Ligands to be docked in the receptor structures were created with ChemBio3D Ultra 13.0 (PerkinElmer, Inc., Waltham, MA, USA) and minimized using the CHARMm force field and Smart Minimizer minimization method of the Minimize Ligands protocol implemented in Biovia Discovery Studio Client (Dassault Systèmes, Vélizy-Villacoublay, France). Prior to starting the molecular docking protocol, ligands were prepared utilizing the Prepare Ligands protocol with regards to possible different protonation states, isomers, and tautomers at pH 7.4. The Flexible Docking protocol [57] was used for docking ligands into the enzyme receptors, with selected residues used to generate enzyme conformations. The protocol consisted of the following steps: enzyme conformation calculation with ChiFlex, ligand conformation creation, ligand docking into active enzyme conformations with LibDock, poses clustering to remove similar ligand poses, enzyme conformation rebuilding by refining selected enzyme side chains in the presence of the rigid ligand with ChiRotor, and final ligand refinement using CDOCKER.

The receptor structures were prepared starting from the crystal structures of free AChE (PDB ID: 4EY4) [25] and BChE (PDB ID: 1P0I) [26]. Next, the network of conserved water molecules identified through superposition of numerous water networks from X-ray structures of various AChE/BChE complexes available from PDB database was mapped onto AChE/BChE structures. The binding site within AChE and BChE model receptors was defined by a sphere (r = 14 Å) surrounding the residues that outline the active sites’ gorge. The following residues were selected as flexible: Tyr72, Trp86, Tyr124, Ser203, Trp286, Phe295, Phe297, Glu334, Tyr337, Phe338, Tyr341, and His447 in the case of AChE and Asn68, Asp70, Trp82, Gln119, Thr120, Ser198, Trp231, Leu286, Val288, Asn289, Glu325, Phe329, Tyr332, Phe398, and His438 in the case of BChE [58,59,60]. For a more detailed description of parameters from the applied docking protocol and pharmacophore modeling study, the reader is referred to the Supplementary Materials.

### 4.4. Cell Tests

#### 4.4.1. Human Cells

Human neuroblastoma SH-SY5Y (ECACC 94030304) and human Caucasian hepatocyte carcinoma HepG2 (ECACC 85011430) were used to evaluate in vitro dose- and time-dependent different cell effects. The SH-SY5Y cell line was selected as it represents a well-established model for studying neuronal cells, while HepG2 hepatocytes represent a liver model as a major organ participating in the metabolism of diverse compounds [31,61,62]. SH-SY5Y cells were grown in DMEM F12 (Sigma-Aldrich, St. Louis, MO, USA) supplemented with 15% (*v*/*v*) fetal bovine serum (FBS), 2 mM glutamine, and 1% (*v*/*v*) non-essential amino acids (NEAA). HepG2 cells were grown in EMEM supplemented with 10% (*v*/*v*) FBS, 2 mM glutamine, and 1% (*v*/*v*) non-essential amino acids NEAA. All media and supplements were purchased from Sigma-Aldrich, Steinheim, Germany.

#### 4.4.2. Cytotoxicity Assay

The 24 h cytotoxic profile of 14 N-alkyl quaternary quinuclidine derivatives was determined with commercially available MTS detection reagent assay (CellTiter 96^®^ AQueous One Solution Cell Proliferation Assay, Promega, Madison, WI, USA). The procedure followed a previously described protocol [63]. The total percentage of DMSO in the assay was 0.8% and did not affect cell viability. Data were evaluated from at least three independent experiments (performed in duplicate or triplicate) and presented as a percentage of the observed cytotoxicity compared to the control untreated cells. IC_50_ values (concentration of compound that kills 50% of cells) were determined by a nonlinear-fit equation predefined in Prism 8 software (GraphPad Software, San Diego, CA, USA). Furthermore, for quinuclidine derivatives that showed a cytotoxic effect in 24 h (namely, derivatives **3**–**5** and **10**–**12**), a more detailed analysis was performed, testing cytotoxicity after 1 h and 4 h exposure. Also, for those derivatives, concentrations corresponding to the Lowest Observed Adverse Effect Level or LOAEL concentrations were determined as the concentration that killed up to ≈ 20–25% of cells in 24 h. Oxime HI-6, a non-toxic known ChE ligand [31], was used as a negative control and staurosporine as a positive control.

#### 4.4.3. Cell Membrane Integrity

Cell membrane integrity was determined after 4 h exposure to derivatives **3**–**5** and **10**–**12** by measuring the release of the intracellular lactate dehydrogenase (LDH), and the procedure followed a previously described protocol [31] using The CytoTox-ONE™ Homogeneous Membrane Integrity Assay (Promega, Madison, WI, USA). Triton X-100 at a final concentration of 0.18% (*v*/*v*) (stock of 9% in water; Sigma-Aldrich, Steinheim, Germany) was used as a positive control to determine the maximal LDH release. Data were taken from at least two independent experiments (each treatment performed in duplicate) and plotted as a percentage of LDH release compared to the determined maximal LDH release, according to the manufacturer’s calculation protocol.

#### 4.4.4. Mitochondrial Membrane Potential

Mitochondrial membrane potential (ΔΨ_m_) was determined using a cell-permeable, potential-sensitive cationic dye, tetramethylrhodamine ethyl ester perchlorate (TMRE, Cell Signaling Technology Europe, Leiden, The Netherlands). Cells were exposed for 4 h to derivatives **3**–**5** and **10**–**12**. The procedure followed a previously described protocol [31]. A concentration of 50 µM carbonyl cyanide 3-chlorophenylhydrazone (CCCP, Cell Signaling Technology Europe, Leiden, The Netherlands) was used as a positive control. Data were evaluated from at least three independent experiments (performed in duplicate) and presented as a normalized signal to the untreated control, according to the manufacturer’s calculation protocol.

#### 4.4.5. Caspases Activity

Caspases (cysteine-aspartic proteases) caspase 3, caspase 8, and caspase 9 were simultaneously analyzed in cells using specific substrate and fluorogenic indicators from the Caspase-3, Caspase-8, and Caspase-9 Multiplex Activity Assay Kit (Abcam, Cambridge, UK). Cells were exposed for 4 h to derivatives **3**–**5** and **10**–**12**. The procedure followed a previously described protocol [31]. A total of 3 µM staurosporine (Sigma-Aldrich, Steinheim, Germany) was used as positive control. Data were evaluated from at least three independent experiments (performed in duplicate or triplicate), according to the manufacturer’s assay kit calculation protocol, and presented as a normalized signal to the untreated control.

### 4.5. Statistics and Calculations

For evaluation and statistical analyses of all experimental data, Prism 8 software (GraphPad Software, San Diego, CA, USA) was used. If not otherwise stated, results are presented as mean and standard error. For statistics, one-way ANOVA followed by Dunnett’s multiple comparisons test was performed to test for differences among groups. Statistical significance is displayed as follows: & *p* ≤ 0.05; # *p* ≤ 0.01; $ *p* ≤ 0.001; * *p* ≤ 0.0001.

## 5. Conclusions

In view of the work already performed in the field of quaternary quinuclidine derivatives, new knowledge on their anticholinesterase activities and cytotoxicity profile is a valuable addition when considering such scaffolds suitable for specific applications or potential therapeutic uses. Here, the tested 14 *N*-alkyl quaternary quinuclidines inhibited both AChE and BChE in the micromolar range, which is in line with currently investigated promising cholinesterase inhibitors of another structural core. The highest influence on the inhibition potency was observed when increasing the alkyl chain length or adding the second quinuclidine ring to their structure. Exactly that, along with the non-cytotoxic profile, made bisquinuclidine alcohol **7** and bisquinuclidine oxime **14** candidates with a therapeutic potential in the cholinergic system worth further investigations. Vice versa, specific cell-related effects probably triggered by the free long alkyl chain in monoquaternary quinuclidine derivatives should not be neglected in future *N*-alkyl quaternary quinuclidine structure refinements. Such effects and their potential to interact with other specific targets, as indicated by a pharmacophore model, open up a new perspective for future investigations of these compounds’ scaffold in the treatment of specific conditions and diseases other than cholinergic system-linked disorders.

## Figures and Tables

**Table 1:** Reversible inhibition of AChE and BChE with *N*-alkyl quaternary quinuclidine alcohols **1**–**7** (OH-C_n_ and bisOH-C_n_) and oximes **8**–**14** (NOH-C_n_ and bisNOH-C_n_) determined in the stated compound concentration range and presented as inhibition constants (*K*_i_).

Compound	Concentration Range (μM)	*K*_i_ (μM)		*K*_i_ (AChE)/*K*_i_ (BChE)
		AChE	BChE	
**1** (OH-C_8_)	200–400	156.2 ± 26.1	85.1 ± 22.1	1.8
**2** (OH-C_10_)	40–60	63.8 ± 5.1	11.8 ± 1.1	5.4
**3** (OH-C_12_)	35–65	13.2 ± 0.6	9.0 ± 0.8	1.5
**4** (OH-C_14_)	2–10	4.2 ± 0.5	7.9 ± 0.7	0.5
**5** (OH-C_16_)	20–40	9.0 ± 1.3	26.1 ± 2.4	0.3
**6** (bisOH-C_8_)	20–40	24.0 ± 1.1	12.9 ± 1.0	1.9
**7** (bisOH-C_10_)	0.2–1	0.52 ± 0.05	1.6 ± 0.2	0.3
**8** (NOH-C_8_)	50–200	82.7 ± 3.5	37.9 ± 10.4	2.2
**9** (NOH-C_10_)	5–50	23.8 ± 0.8	5.2 ± 1.1	4.6
**10** (NOH-C_12_)	5–15	14.3 ± 1.8	5.4 ± 0.8	2.6
**11** (NOH-C_14_)	5–10	7.0 ± 0.9	8.8 ± 1.0	0.8
**12** (NOH-C_16_)	40–60	19.0 ± 1.6	48.6 ± 3.8	0.4
**13** (bisNOH-C_8_)	5–20	1.8 ± 0.07	3.4 ± 0.2	0.5
**14** (bisNOH-C_10_)	0.2–2	0.26 ± 0.02	1.2 ± 0.06	0.2

The reversible enzyme–quinuclidine complex dissociation constant or the inhibition constants (*K*_i_ ± standard error) were determined from at least three experiments at 0.1, 0.2, 0.35, and 0.5 mM substrate ATCh.

**Table 2:** Cytotoxicity (IC_50_ values) of tested *N*-alkyl quaternary quinuclidine alcohols **1**–**7** (OH-C_n_ and bisOH-C_n_) and oximes **8**–**14** (NOH-C_n_ and bisNOH-C_n_) after 1, 4, and 24 h of exposure of SH-SY5Y and HepG2 cells.

	IC_50_ ± SE (μM)					
Compound	SH-SY5Y			HepG2		
	1 h	4 h	24 h	1 h	4 h	24 h
**1** (OH-C_8_)	≥800	≥800	≥800	≥800	≥800	≥800
**2** (OH-C_10_)	≥800	≥800	≥800	≥800	≥800	≥537
**3** (OH-C_12_)	145 ± 1	74 ± 1	56 ± 1	135 ± 1	81 ± 1	66 ± 1
**4** (OH-C_14_)	16 ± 1	9 ± 1	7 ± 1	35 ± 1	32 ± 1	43 ± 1
**5** (OH-C_16_)	11 ± 1	8 ± 1	7 ± 1	46 ± 1	26 ± 1	28 ± 1
**6** (bisOH-C_8_)	≥800	≥800	≥800	≥800	≥800	≥800
**7** (bisOH-C_10_)	≥800	≥800	≥800	≥800	≥800	≥800
**8** (NOH-C_8_)	≥800	≥800	≥800	≥800	≥800	≥800
**9** (NOH-C_10_)	≥800	≥800	≥800	≥800	≥800	≥800
**10** (NOH-C_12_)	208 ± 3	174 ± 1	147 ± 1 ^a^	218 ± 1	81 ± 1	15 ± 1
**11** (NOH-C_14_)	125 ± 2	83 ± 3	60 ± 1	37 ± 1	18 ± 1	11 ± 1
**12** (NOH-C_16_)	89 ± 1	78 ± 1	54 ± 1	40 ± 1	20 ± 1	7 ±1
**13** (bisNOH-C_8_)	≥800	≥800	≥800	≥800	≥800	≥800
**14** (bisNOH-C_10_)	≥800	≥800	≥800	≥800	≥800	≥800
HI-6 ^b^	≥800	≥800	≥800	≥800	≥800	≥800
SS ^b^	≥4	3 ± 0.01	0.12 ± 0.01	≥2	≥2	≥2

^a^ from [30]; ^b^ HI-6—pyridinium oxime used as a negative control; SS (staurosporine)—positive control; from [31].

## Data Availability

All data are available in the manuscript and within Supplementary Materials.

## Supplementary Materials

The following supporting information can be downloaded at: https://www.mdpi.com/article/10.3390/ijms25010155/s1.
