# Supplementary material for: Profiling Novel Quinuclidine-Based Derivatives as Potential Anticholinesterase Drugs: Enzyme Inhibition and Effects on Cell Viability
Source: Int J Mol Sci. 2023 Dec 21;25(1):155. doi: 10.3390/ijms25010155 (PMC10778980; doi:10.3390/ijms25010155)
Supplement: Supplementary file 1 [file ijms-25-00155-s001.zip › ijms-2764509-supplementary.pdf]

Supplementary Material

Profiling novel quinuclidine based derivatives as potential anticholinesterase drugs: enzyme inhibition and effects on cell viability

Suzana Žunec[1], Donna Vadlja[2], Antonio Zandona[1], Nikola Maraković[1], Alma Ramić[3], Ines Primožič[3] and Maja Katalinić[1,*]

[1]Division of Toxicology, Institute for Medical Research and Occupational Health, Ksaverska c. 2, 10 000 Zagreb, Croatia; suzana@imi.hr (S.Ž.); azandona@imi.hr (A.Z.); nmarakovic@imi.hr (N.M.)

[2]Armed Forces of the Republic of Croatia, Trg kralja Petra Krešimira IV br. 1, 10000 Zagreb, Croatia; donna.vadlja@gmail.com

[3]Faculty of Science, University of Zagreb, Horvatovac 102a, 10000 Zagreb, Croatia; ar-amic@chem.pmf.hr (A.R.); ines.primozic@pmf.hr (I.P.)

*Correspondence: mkatalinic@imi.hr; Tel.: +385-1-4682-551

Content:

- 1H and 13C NMR spectra of novel compounds
  Figure S1 – S12

- Detailed description of docking procedure
  List of interactions between tested *N*-alkyl quaternary quinuclidines and AChE
  Table S1-S14
  List of interactions between tested *N*-alkyl quaternary quinuclidines and BChE
  Table S15-S28

- The cytotoxicity of the selected *N*-alkyl quaternary quinuclidines
  Figure S13 and S14

- Pharmacophore modeling and *in silico* prediction of targets
  Figure S15

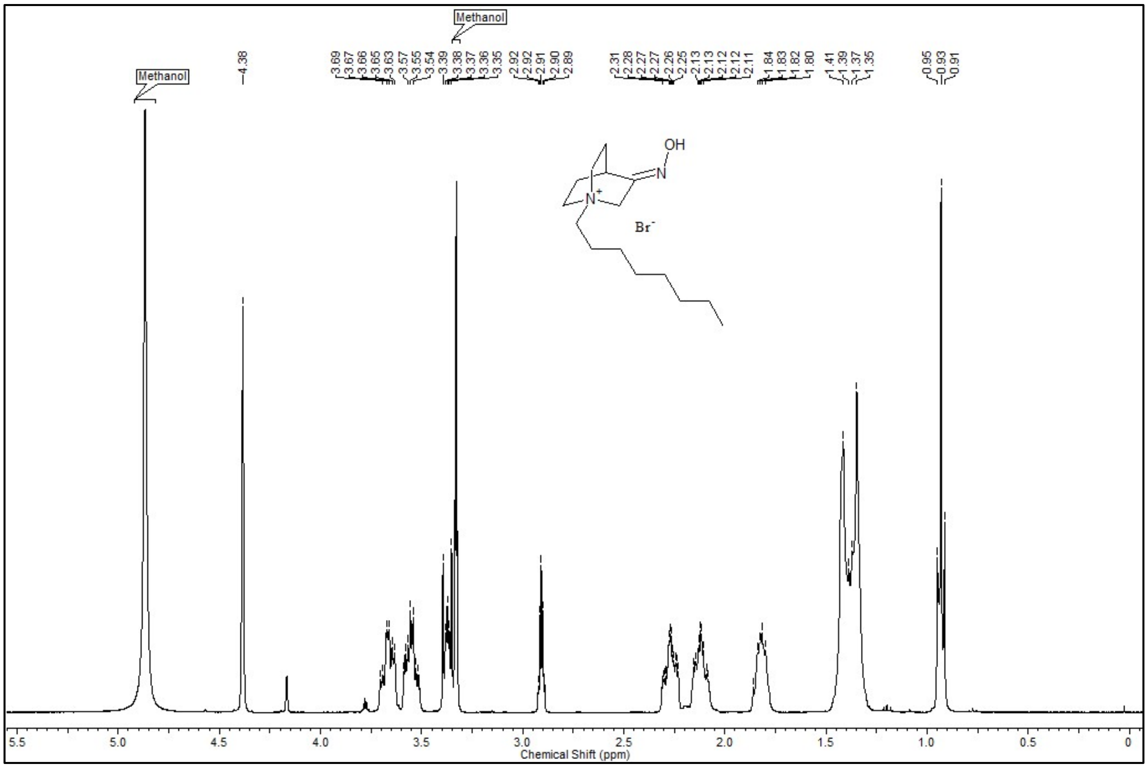

Figure S1. ^{1}H NMR spectrum of compound **8**

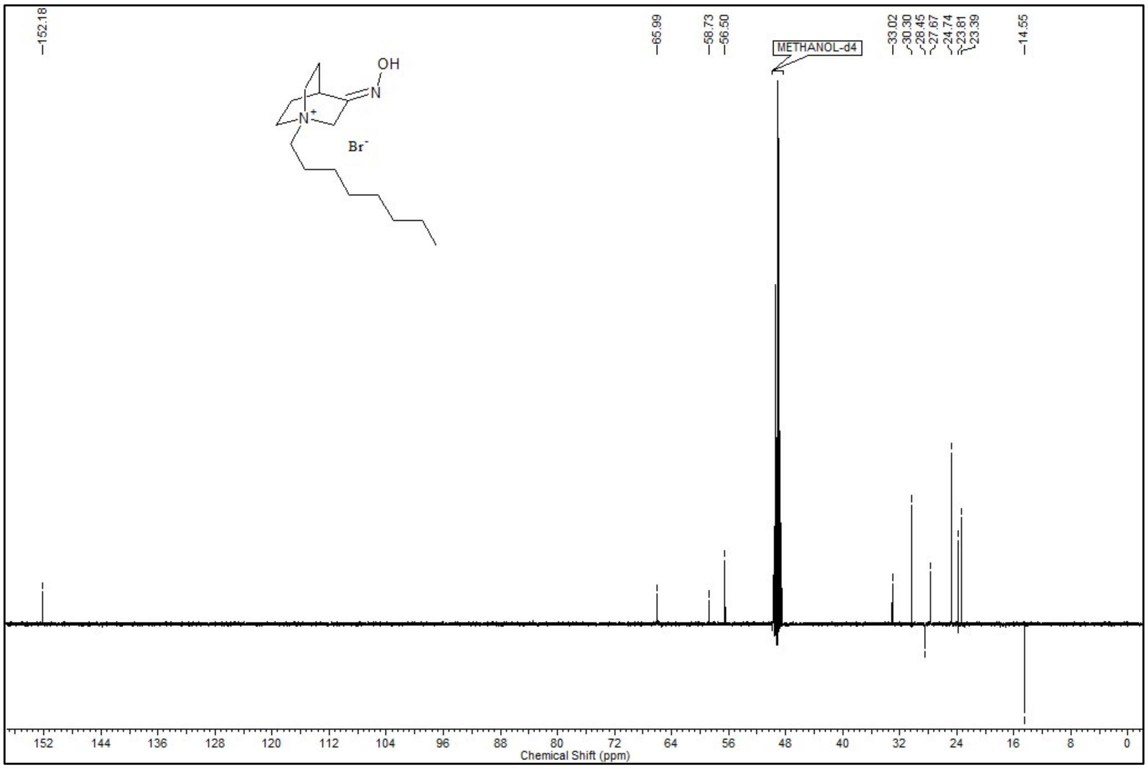

Figure S2. ^{13}C NMR spectrum of compound **8**

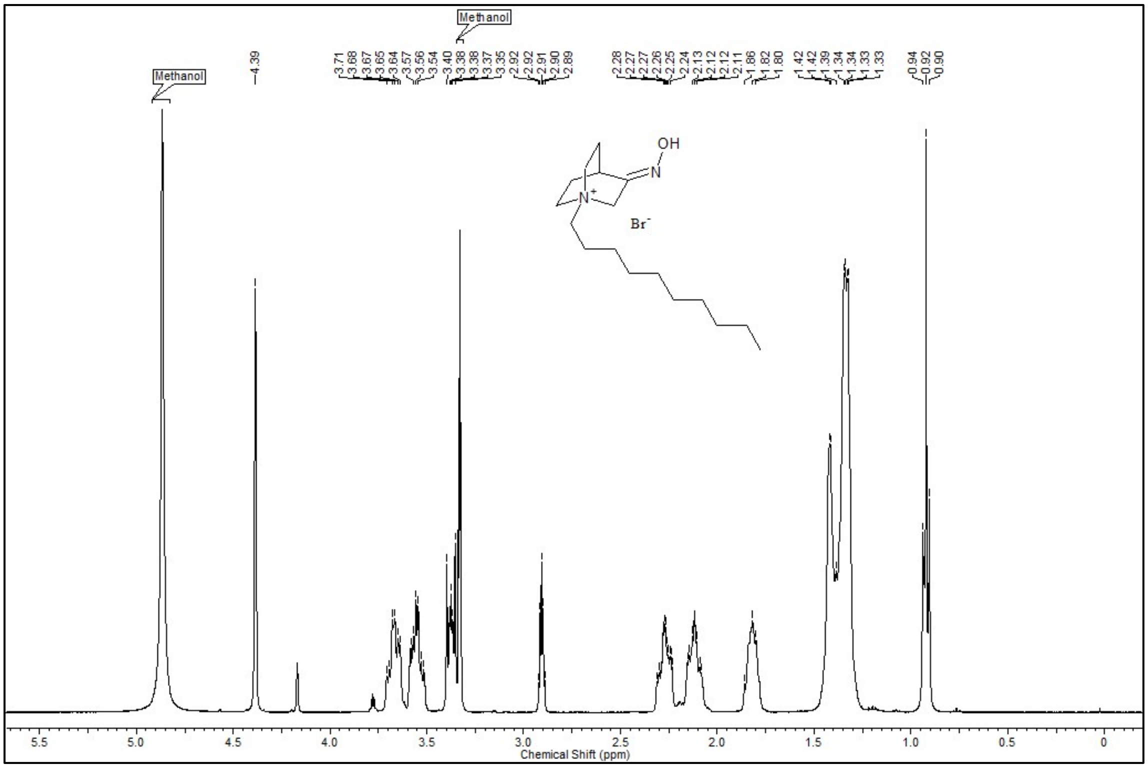

Figure S3. ^{1}H NMR spectrum of compound **9**

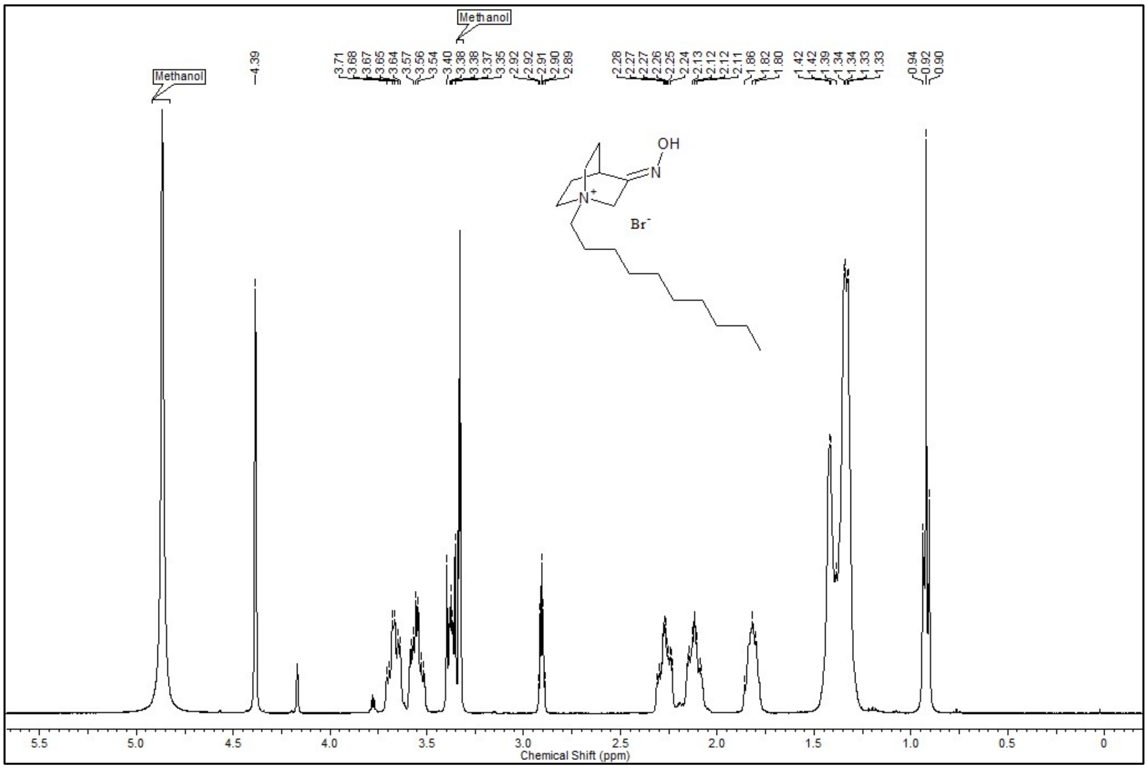

Figure S4. ^{13}C NMR spectrum of compound **9**

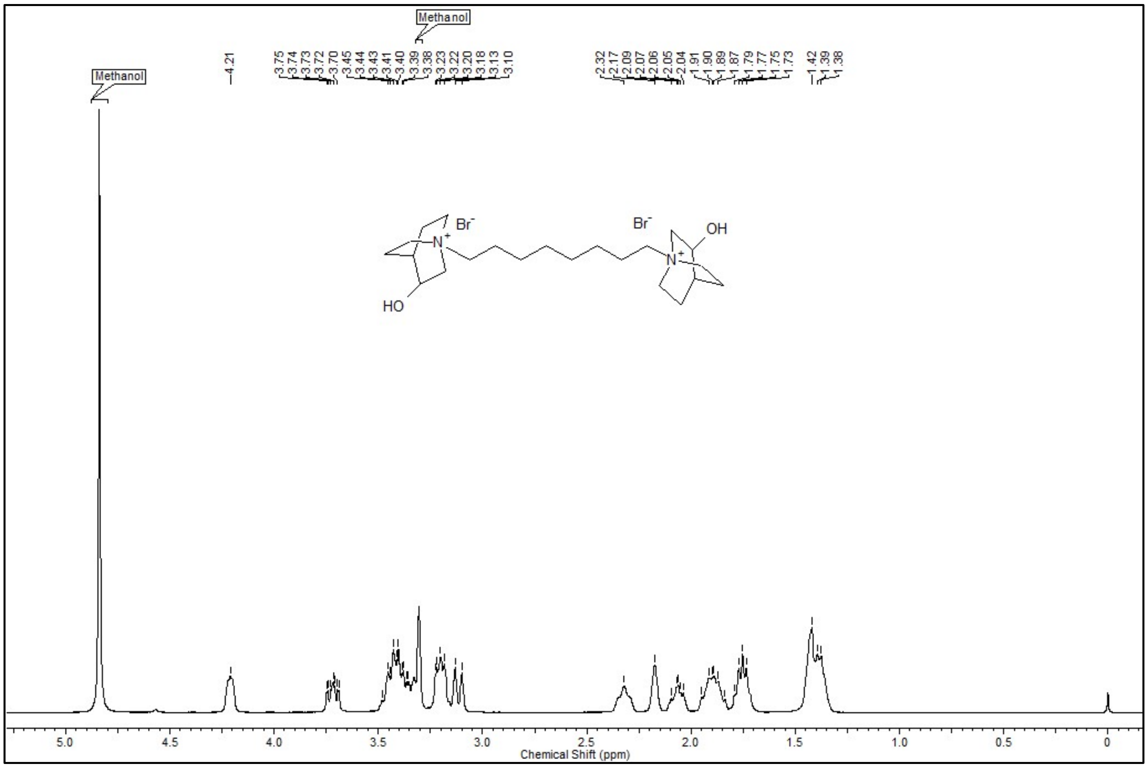

Figure S5. ^{1}H NMR spectrum of compound **6**

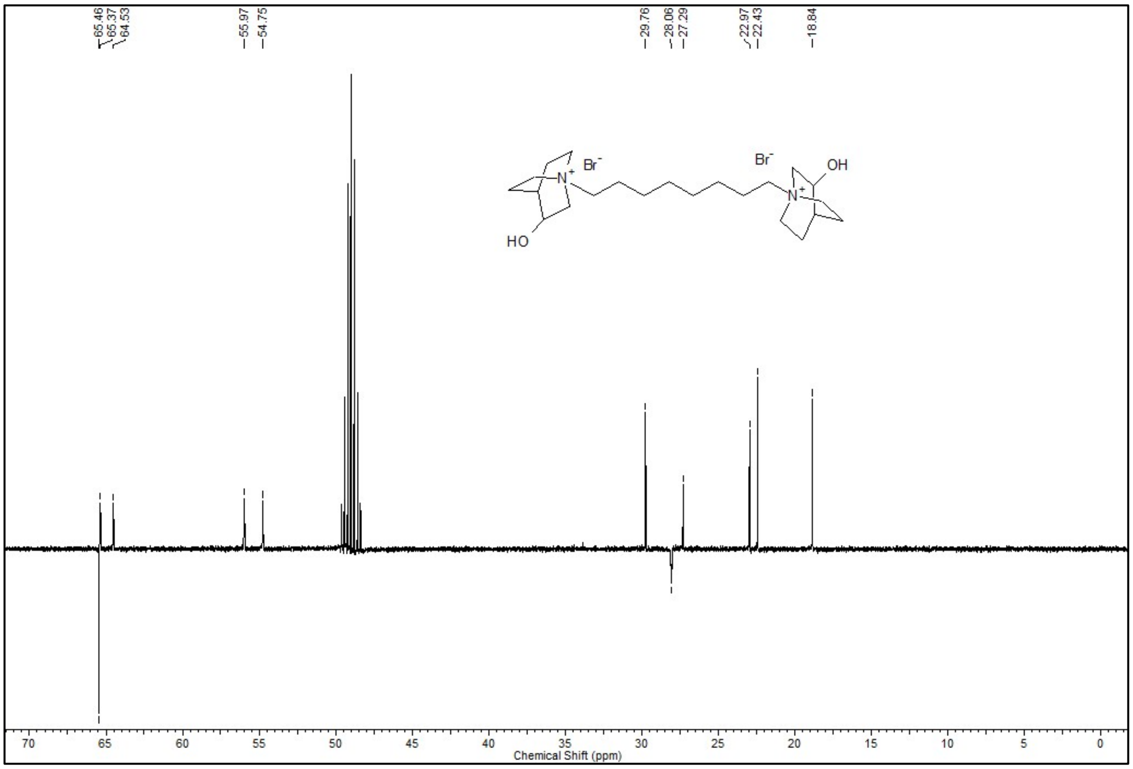

Figure S6. ^{13}C NMR spectrum of compound **6**

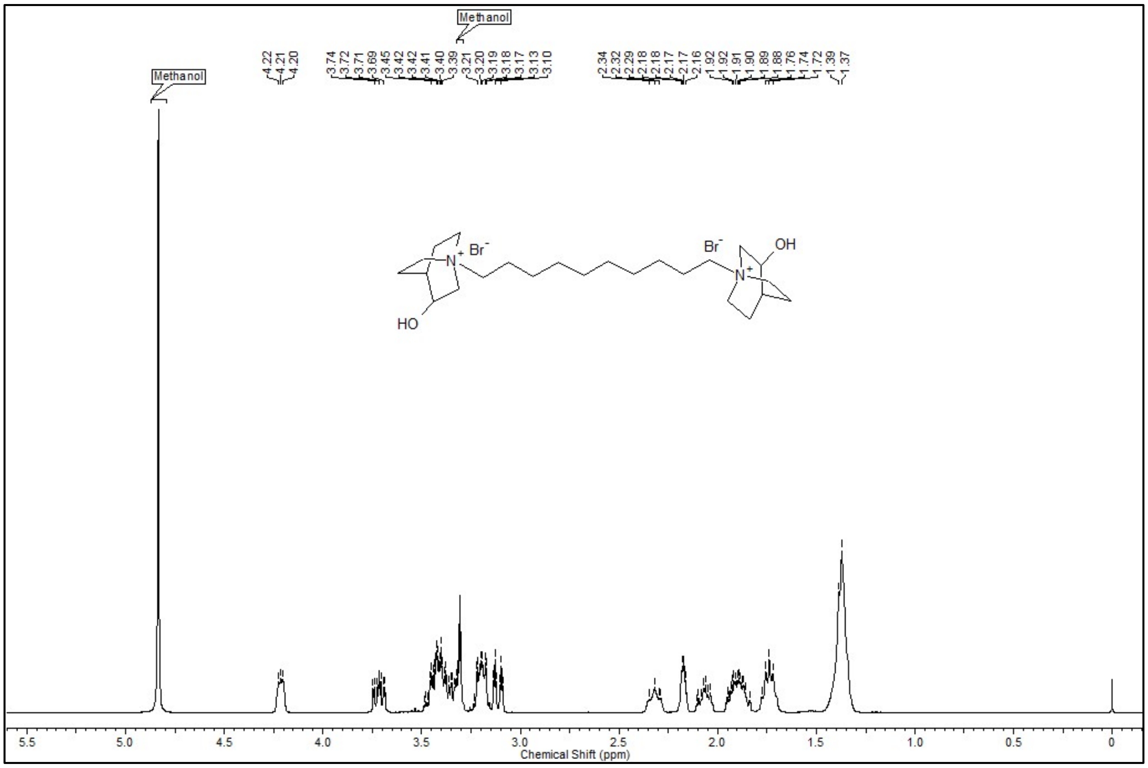

Figure S7. ^{1}H NMR spectrum of compound **7**

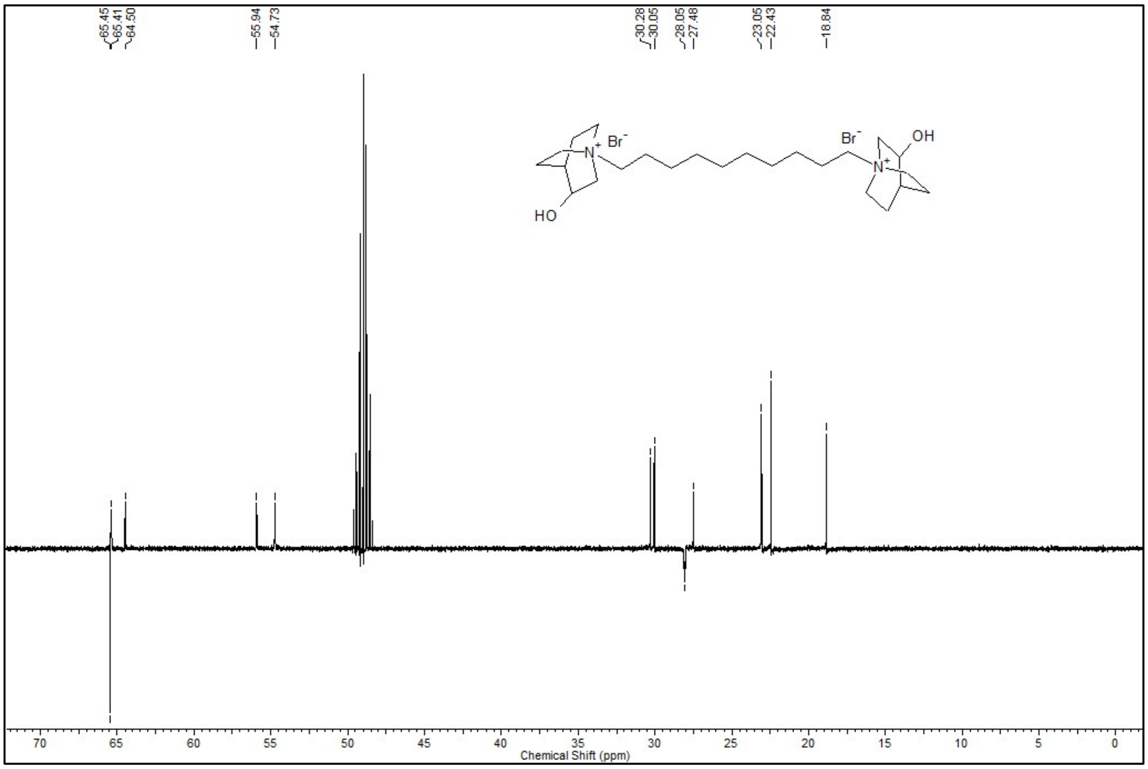

Figure S8. ^{13}C NMR spectrum of compound **7**

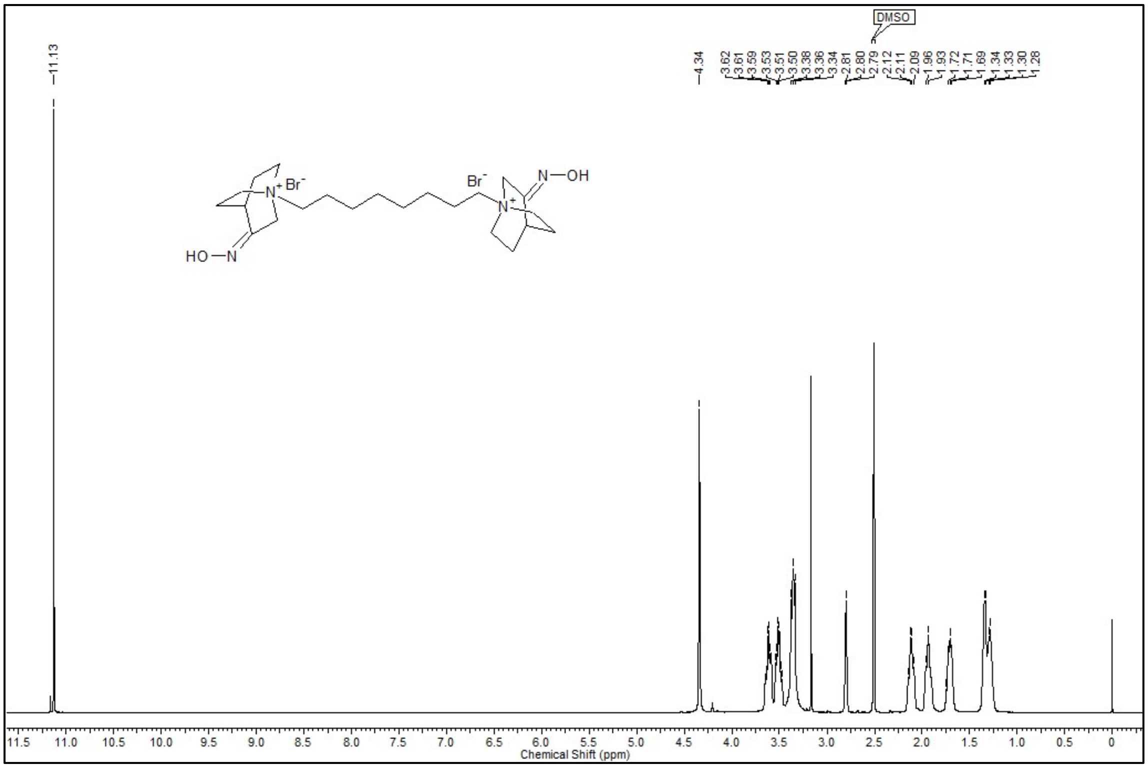

Figure S9. ^{1}H NMR spectrum of compound **13**

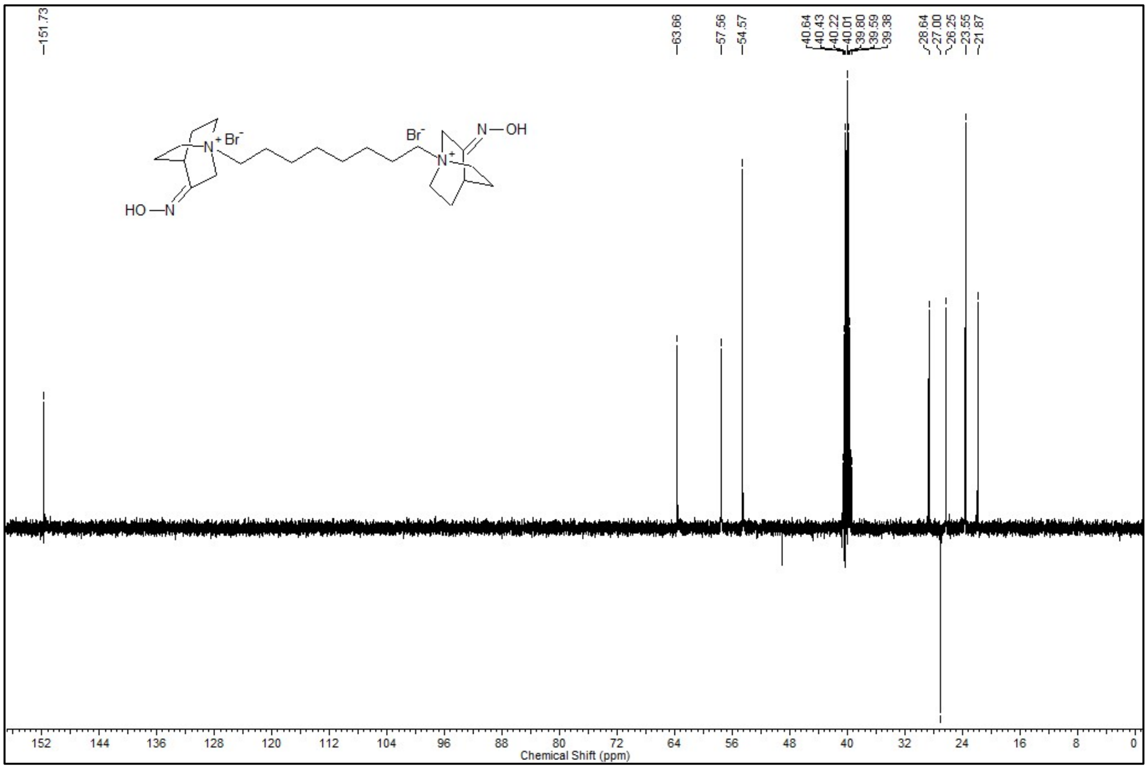

Figure S10. ^{13}C NMR spectrum of compound **13**

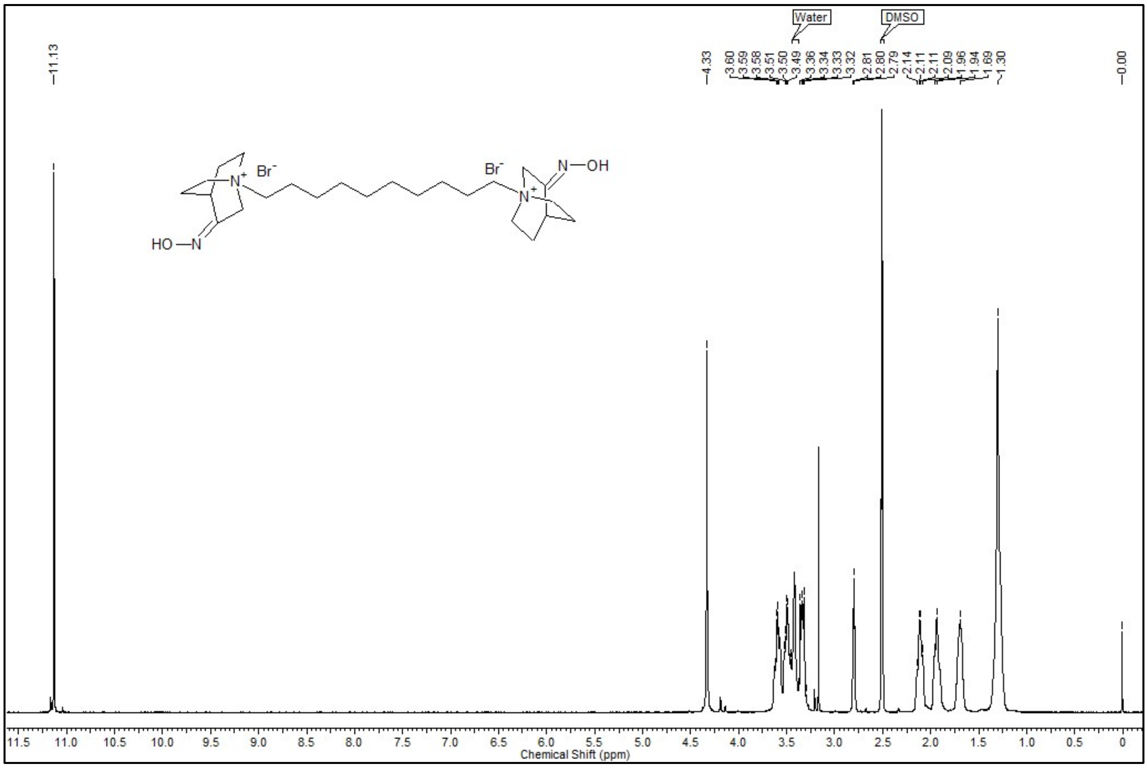

Figure S11. ^{1}H NMR spectrum of compound **14**

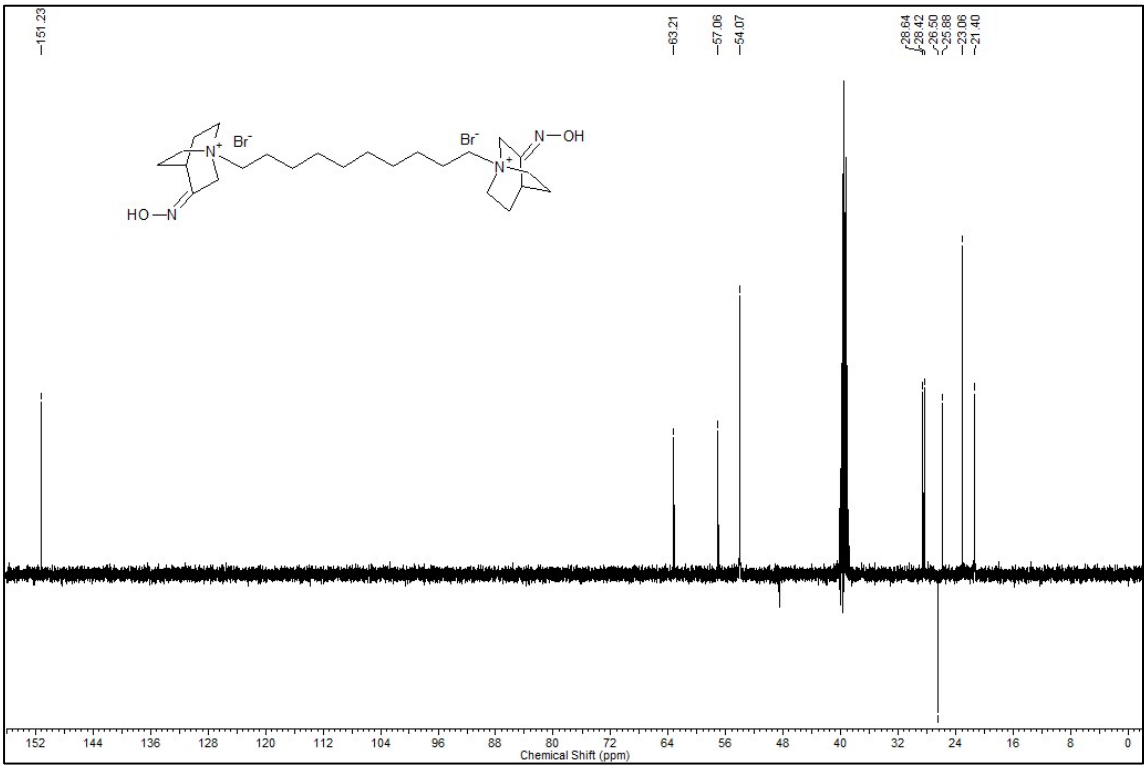

Figure S12. ^{13}C NMR spectrum of compound **14**

Detailed description of docking procedure

The parameters from the Flexible Docking protocol were set as follows: Maximum number of enzyme conformations processed for ligand docking was set to unlimited; Minimum angel, i.e. torsion angle cutoff (degrees) to determine if two side-chain chi1 angles are the same or not, was set to 15; Conformation Method, i.e. algorithm for generating ligand conformations, was set to BEST; Maximum number of conformations to be generated was set to 255; Energy Threshold, i.e. relative energy threshold (kcal/mol) within which conformations of separate isomers are created, was set to 20. Parameters for LibDock ligand docking were set as follows: Number of Hotspots, i.e. number of polar or apolar receptor hotspots for conformer matching, and Max Hits to Save were set to 100 and 30, respectively. Tolerance for a hotspots matching algorithm to dock ligands was set to 0.25; Maximum Number of Hits which specifies the maximum number of hits saved for each ligand during hotspots matching before the final pose minimization was set to 100 with Final Score Cutoff, i.e. fraction of the top scoring poses reported set to 0.5; Maximum number of poses to be kept per conformation (i.e. Max Conformation Hits) was set to 30 with Maximum number of conformations for each ligand (i.e. Max Start Conformations) set to 1000; Steric Fraction defining number of clashes before the pose-hotspot alignment is terminated was set to 0.10 within the 0.5 Å Final Cluster Radius; Apolar SASA Cutoff and Polar SASA Cutoff values were set to 15.0 Å and 5.0 Å, respectively. Finally, simulated annealing refinement was performed with 2000 heating phase steps during simulated annealing, 5000 steps of the cooling phase, and Heating Target Temperature and Cooling Target Temperature set to 700 K and 310 K, respectively.

The representative pose of each of the docked ligands was chosen based on the highest score as predicted by the scoring functions estimating binding affinities, implemented in the Biovia Discovery Studio Client. Score Ligand Poses protocol. The following scoring functions were calculated: CDOCKER Scores, LigScore1, LigScore2, PLP1, PLP2, Jain, PMF, PMF04, Ludi Energy Estimate 1, Ludi Energy Estimate 2, and Ludi Energy Estimate 3. To identify the poses of docked ligands that score high in more than one scoring function, the Biovia Discovery Studio Client Consensus Score protocol was used. The Consensus Score protocol calculates the consensus scores of a series of docked ligands for which other scores have been previously computed. For each selected scoring function, the ligands are listed by score in descending order. The consensus score for a ligand is an integer between zero (none of the scores are in the top-ranking percentile) and the total number of scores (all of the scores are in the top-ranking percentile) listed in Input

Properties. Thus, in the Parameters Explorer of Consensus Score protocol, following scoring functions were chosen to calculate the consensus score: -CDOCKER_ENERGY, -CDOCKER_INTERACTION_ENERGY, LigScore1, LigScore2, PLP1, PLP2, Jain, PMF, PMF04, Ludi Energy Estimate 1, Ludi Energy Estimate 2, and Ludi Energy Estimate 3. Consensus Percentage was set to 20 to specify the percentage of top molecules to include in the consensus. Use Best Pose only was set to False. Finally, the poses with highest consensus score were visually inspected and those predicting binding motifs typical of AChE/BChE complexes were chosen as the representative poses of each of the docked ligands.

List of interactions between tested *N*-alkyl quaternary quinuclidines and AChE

Table S1. Interactions between compound (*R*)-QOH-C_8 and AChE.

Amino acid	Non-bonding interactions
Ser203	O9, hydrogen bond-conventional hydrogen bond
Trp86	N1, electrostatic-π-cation
Trp82	H24, hydrophobic-π-sigma
Tyr124	C10, hydrophobic-π-alkyl
Trp286	C10, hydrophobic-π-alkyl
Tyr337	R-C8, hydrophobic-π-alkyl
Phe338	R-C8, hydrophobic-π-alkyl
Tyr341	C10, hydrophobic-π-alkyl

Table S2. Interactions between compound (*S*)-QOH-C_{10} and AChE.

Amino acid	Non-bonding interactions
HOH752	O9, hydrogen bond-water hydrogen bond; conventional hydrogen bond
Glu202	H32, hydrogen bond-conventional hydrogen bond
Trp86	N1, electrostatic-π-cation
Tyr72	C10, hydrophobic-π-alkyl
Trp286	C10, hydrophobic-π-alkyl
Trp286	C10, hydrophobic-π-alkyl
Trp286	S-C10, hydrophobic-π-alkyl
Tyr337	S-C10, hydrophobic-π-alkyl
Phe338	S-C10, hydrophobic-π-alkyl
Tyr341	S-C10, hydrophobic-π-alkyl
Tyr341	S-C10, hydrophobic-π-alkyl

Table S3. Interactions between compound (*R*)-QOH-C_{12} and AChE.

Amino acid	Non-bonding interactions
Glu202	N1, electrostatic-attractive charge
His447	H34, hydrogen bond-conventional hydrogen bond
Glu202	H26, hydrogen bond-carbon hydrogen bond
Glu202	H33, hydrogen bond-carbon hydrogen bond
Ser203	H58, hydrogen bond-carbon hydrogen bond
Leu76	C10, hydrophobic-alkyl
Val340	C10, hydrophobic-alkyl

Tyr124	R-C12, hydrophobic-π-alkyl
Phe297	R-C12, hydrophobic-π-alkyl
Tyr337	C10, hydrophobic-π-alkyl
Phe338	R-C12, hydrophobic-π-alkyl
Tyr341	R-C12, hydrophobic-π-alkyl
Trp439	R-C12, hydrophobic-π-alkyl

Table S4. Interactions between compound (*S*)-QOH-C_{14} and AChE.

Amino acid	Non-bonding interactions
Glu202	N1, electrostatic-attractive charge
Gly448	O9, hydrogen bond-carbon hydrogen bond
Trp86	N1, electrostatic-π-cation
Trp86	N1, electrostatic-π-cation
Leu289	C10, hydrophobic-alkyl
Trp286	C10, hydrophobic-π-alkyl
Trp286	S-C14, hydrophobic-π-alkyl
Trp286	C10, hydrophobic-π-alkyl
Trp286	S-C14, hydrophobic-π-alkyl
Phe297	S-C14, hydrophobic-π-alkyl
Tyr337	S-C14, hydrophobic-π-alkyl
Tyr337	S-C14, hydrophobic-π-alkyl
Phe338	S-C14, hydrophobic-π-alkyl
Tyr341	S-C14, hydrophobic-π-alkyl
Tyr341	S-C14, hydrophobic-π-alkyl

Table S5. Interactions between compound (*S*)-QOH-C_{16} and AChE.

Amino acid	Non-bonding interactions
Glu202	H38, hydrogen bond-conventional hydrogen bond
His447	O9, hydrogen bond-carbon hydrogen bond
Gly448	O9, hydrogen bond-carbon hydrogen bond
Tyr337	H70, hydrogen bond-carbon hydrogen bond
Trp86	N1, electrostatic-π-cation
Tyr72	C10, hydrophobic-π-alkyl
Tyr72	S-C16, hydrophobic-π-alkyl
Tyr72	S-C16, hydrophobic-π-alkyl
Tyr124	S-C16, hydrophobic-π-alkyl
Trp286	C10, hydrophobic-π-alkyl
Trp286	S-C16, hydrophobic-π-alkyl
Trp286	C10, hydrophobic-π-alkyl
Trp286	S-C16, hydrophobic-π-alkyl
Tyr337	S-C16, hydrophobic-π-alkyl
Phe338	S-C16, hydrophobic-π-alkyl
Tyr341	S-C16, hydrophobic-π-alkyl
Tyr341	S-C16, hydrophobic-π-alkyl

Table S6. Interactions between compound (*R*,*R*)-bisQOH-C_8 and AChE.

Amino acid	Non-bonding interactions
Asp74	N24, electrostatic-attractive charge
His447	O25, hydrogen bond-conventional hydrogen bond
Gly448	O25, hydrogen bond-carbon hydrogen bond
Tyr124	H65, hydrogen bond-carbon hydrogen bond

Tyr124	N24, electrostatic-π-cation
Tyr124	H42, hydrophobic-π-sigma
Tyr124	R,R-DI-C8, hydrophobic-π-alkyl
Tyr337	R,R-DI-C8, hydrophobic-π-alkyl
Tyr341	R,R-DI-C8, hydrophobic-π-alkyl

Table S7. Interactions between compound (*R,S*)-bisQOH-C_{10} and AChE.

Amino acid	Non-bonding interactions
Glu202	N1, electrostatic-attractive charge
His447	O27, hydrogen bond-conventional hydrogen bond
Gly448	O27, hydrogen bond-carbon hydrogen bond
Ser203	H53, hydrogen bond-carbon hydrogen bond
Tyr72	N24, electrostatic-π-cation
Tyr337	R,S-DI-C10, hydrophobic-π-alkyl
Phe338	R,S-DI-C10, hydrophobic-π-alkyl
Tyr341	R,S-DI-C10, hydrophobic-π-alkyl

Table S8. Interactions between compound QNOH-C_8 and AChE.

Amino acid	Non-bonding interactions
HOH772	N9, hydrogen bond-water hydrogen bond; conventional hydrogen bond
Trp86	N1, electrostatic-π-cation
Tyr124	OX-C8, hydrophobic-π-alkyl
Trp236	C11, hydrophobic-π-alkyl
Trp236	C11, hydrophobic-π-alkyl
Phe295	C11, hydrophobic-π-alkyl
Phe297	OX-C8, hydrophobic-π-alkyl
Phe338	OX-C8, hydrophobic-π-alkyl
His447	C11, hydrophobic-π-alkyl

Table S9. Interactions between compound QNOH-C_{10} and AChE.

Amino acid	Non-bonding interactions
HOH784	N9, hydrogen bond-water hydrogen bond; conventional hydrogen bond
Trp286	C11, hydrophobic-π-alkyl
Trp286	C11, hydrophobic-π-alkyl
Trp286	OX-C10, hydrophobic-π-alkyl
Tyr341	OX-C10, hydrophobic-π-alkyl
Tyr341	OX-C10, hydrophobic-π-alkyl

Table S10. Interactions between compound QNOH-C_{12} and AChE.

Amino acid	Non-bonding interactions
His447	H34, hydrogen bond-conventional hydrogen bond
Tyr124	OX-C12, hydrophobic-π-alkyl
Trp286	OX-C12, hydrophobic-π-alkyl
Tyr337	OX-C12, hydrophobic-π-alkyl
Phe338	OX-C12, hydrophobic-π-alkyl
Tyr341	OX-C12, hydrophobic-π-alkyl
Tyr341	OX-C12, hydrophobic-π-alkyl

Table S11. Interactions between compound QNOH-C_{14} and AChE.

Amino acid	Non-bonding interactions
HOH752	N9, hydrogen bond-water hydrogen bond; conventional hydrogen bond
HOH784	N9, hydrogen bond-water hydrogen bond; conventional hydrogen bond
Glu120	H36, hydrogen bond-conventional hydrogen bond
Tyr124	OX-C14, hydrophobic-π-alkyl
Trp286	C11, hydrophobic-π-alkyl
Trp286	OX-C14, hydrophobic-π-alkyl
Trp286	OX-C14, hydrophobic-π-alkyl
Trp286	OX-C14, hydrophobic-π-alkyl
His287	C11, hydrophobic-π-alkyl
Phe338	OX-C14, hydrophobic-π-alkyl
Tyr341	OX-C14, hydrophobic-π-alkyl
Tyr341	OX-C14, hydrophobic-π-alkyl

Table S12. Interactions between compound QNOH-C_{16} and AChE.

Amino acid	Non-bonding interactions
Leu76	C11, hydrophobic-alkyl
Leu76	OX-C16, hydrophobic-alkyl
Tyr72	OX-C16, hydrophobic-π-alkyl
Tyr124	OX-C16, hydrophobic-π-alkyl
Trp286	OX-C16, hydrophobic-π-alkyl
Trp286	OX-C16, hydrophobic-π-alkyl
Trp286	OX-C16, hydrophobic-π-alkyl
Phe338	OX-C16, hydrophobic-π-alkyl
Tyr341	OX-C16, hydrophobic-π-alkyl

Table S13. Interactions between compound bisQNOH-C_{8} and AChE.

Amino acid	Non-bonding interactions
HOH752	N18, hydrogen bond-water hydrogen bond; conventional hydrogen bond
Glu120	H52, hydrogen bond-conventional hydrogen bond
Tyr124	N1, electrostatic-π-cation
Trp86	N28, electrostatic-π-cation
Tyr72	H36, hydrophobic-π-sigma
Tyr341	OX-BIS-C8, hydrophobic-π-alkyl

Table S14. Interactions between compound bisQNOH-C_{10} and AChE.

Amino acid	Non-bonding interactions
HOH752	N9, hydrogen bond-water hydrogen bond; conventional hydrogen bond
Glu120	H42, hydrogen bond-conventional hydrogen bond
Tyr72	H43, hydrogen bond-carbon hydrogen bond
Trp86	N1, electrostatic-π-cation
Tyr124	OX-BIS-C10, hydrophobic-π-alkyl
Trp286	OX-BIS-C10, hydrophobic-π-alkyl
Tyr337	OX-BIS-C10, hydrophobic-π-alkyl
Phe338	OX-BIS-C10, hydrophobic-π-alkyl
Tyr341	OX-BIS-C10, hydrophobic-π-alkyl
Tyr341	OX-BIS-C10, hydrophobic-π-alkyl

List of interactions between tested *N*-alkyl quaternary quinuclidines and BChE

Table S15. Interactions between compound (*R*)-QOH-C_8 and BChE.

Amino acid	Non-bonding interactions
Glu197	N1, electrostatic-attractive charge
HOH705	O9, hydrogen bond-water hydrogen bond; conventional hydrogen bond
HOH709	H19, hydrogen bond-water hydrogen bond; carbon hydrogen bond
HOH708	H30, hydrogen bond-water hydrogen bond; conventional hydrogen bond
Trp82	N1, electrostatic-π-cation
Trp82	N1, electrostatic-π-cation
Trp82	H29, hydrophobic-π-sigma
Leu286	C10, hydrophobic-alkyl
Val288	C10, hydrophobic-alkyl
Trp231	C10, hydrophobic-π-alkyl
Trp231	C10, hydrophobic-π-alkyl
Phe329	R-C8, hydrophobic-π-alkyl

Table S16. Interactions between compound (*S*)-QOH-C_{10} and BChE.

Amino acid	Non-bonding interactions
Glu197	N1, electrostatic-attractive charge
HOH786	O9, hydrogen bond-water hydrogen bond; conventional hydrogen bond
HOH709	H23, hydrogen bond-water hydrogen bond; carbon hydrogen bond
Glu197	H32, hydrogen bond-conventional hydrogen bond
His438	O9, hydrogen bond-carbon hydrogen bond
Trp82	N1, electrostatic-π-cation
Leu286	C10, hydrophobic-alkyl
Trp231	C10, hydrophobic-π-alkyl
Trp231	C10, hydrophobic-π-alkyl
Phe398	C10, hydrophobic-π-alkyl

Table S17. Interactions between compound (*R*)-QOH-C_{12} and BChE.

Amino acid	Non-bonding interactions
Glu197	N1, electrostatic-attractive charge
Trp82	N1, electrostatic-π-cation
Trp82	N1, electrostatic-π-cation
Leu286	C10, hydrophobic-alkyl
Val288	C10, hydrophobic-alkyl
Trp231	C10, hydrophobic-π-alkyl
Trp231	C10, hydrophobic-π-alkyl
Tyr332	S-C12, hydrophobic-π-alkyl

Table S18. Interactions between compound (*S*)-QOH-C_{14} and BChE.

Amino acid	Non-bonding interactions
Glu197	N1, electrostatic-attractive charge
Trp82	N1, electrostatic-π-cation
Trp82	N1, electrostatic-π-cation
Leu286	C10, hydrophobic-alkyl
Val288	C10, hydrophobic-alkyl
Trp231	C10, hydrophobic-π-alkyl

Trp231	C10, hydrophobic-π-alkyl

Table S19. Interactions between compound (*S*)-QOH-C_{16} and BChE.

Amino acid	Non-bonding interactions
Glu197	N1, electrostatic-attractive charge
Gly439	O9, hydrogen bond-carbon hydrogen bond
Glu197	H26, hydrogen bond-carbon hydrogen bond
Glu197	H29, hydrogen bond-carbon hydrogen bond
Val288	S-C16, hydrophobic-alkyl
Leu286	S-C16, hydrophobic-alkyl
Trp231	C10, hydrophobic-π-alkyl
Trp231	C10, hydrophobic-π-alkyl
Phe329	S-C16, hydrophobic-π-alkyl
Phe398	C10, hydrophobic-π-alkyl

Table S20. Interactions between compound (*R,R*)-bisQOH-C_8 and BChE.

Amino acid	Non-bonding interactions
Glu197	N1, electrostatic-attractive charge
HOH1031	O26, hydrogen bond-water hydrogen bond; conventional hydrogen bond
HOH706	O25, hydrogen bond-water hydrogen bond; conventional hydrogen bond
HOH1031	H45, hydrogen bond-water hydrogen bond; carbon hydrogen bond
HOH930	H66, hydrogen bond-water hydrogen bond; carbon hydrogen bond
Ser198	O25, hydrogen bond-conventional hydrogen bond
Glu197	H67, hydrogen bond-conventional hydrogen bond
His438	H67, hydrogen bond-conventional hydrogen bond
Gln119	H41, hydrogen bond-carbon hydrogen bond
Gln119	H42, hydrogen bond-carbon hydrogen bond
Trp82	N1, electrostatic-π-cation
Trp82	N1, electrostatic-π-cation
Tyr332	R,R-DI-C8, hydrophobic-π-alkyl

Table S21. Interactions between compound (*R,S*)-bisQOH-C_{10} and BChE.

Amino acid	Non-bonding interactions
Glu197	N24, electrostatic-attractive charge
HOH884	O27, hydrogen bond-water hydrogen bond; conventional hydrogen bond
Gly439	O28, hydrogen bond-carbon hydrogen bond
Pro285	H31, hydrogen bond-carbon hydrogen bond
Gn119	H32, hydrogen bond-carbon hydrogen bond
Trp82	N24, electrostatic-π-cation
Ala328	R,S-DI-C10, hydrophobic-alkyl
Phe329	R,S-DI-C10, hydrophobic-π-alkyl
Tyr332	R,S-DI-C10, hydrophobic-π-alkyl

Table S22. Interactions between compound QNOH-C_8 and BChE.

Amino acid	Non-bonding interactions
HOH706	N9, hydrogen bond-water hydrogen bond; conventional hydrogen bond
HOH708	H30, hydrogen bond-water hydrogen bond; conventional hydrogen bond
Glu197	H30, hydrogen bond-conventional hydrogen bond
Gly115	O10, hydrogen bond-carbon hydrogen bond

Ser198	N9, hydrogen bond-carbon hydrogen bond
Ser198	N9, hydrogen bond-carbon hydrogen bond
Leu286	C11, hydrophobic-alkyl
Trp231	C11, hydrophobic-π-alkyl
Trp231	C11, hydrophobic-π-alkyl
Phe329	OX-C8, hydrophobic-π-alkyl

Table S23. Interactions between compound QNOH-C_{10} and BChE.

Amino acid	Non-bonding interactions
Ser198	H32, hydrogen bond-conventional hydrogen bond
His438	H32, hydrogen bond-conventional hydrogen bond
Ser198	N9, hydrogen bond-carbon hydrogen bond
Trp82	N1, electrostatic-π-cation
Trp82	H24, hydrophobic-π-sigma
Leu286	C11, hydrophobic-alkyl
Trp231	C11, hydrophobic-π-alkyl
Trp231	C11, hydrophobic-π-alkyl
Tyr332	OX-C10, hydrophobic-π-alkyl

Table S24. Interactions between compound QNOH-C_{12} and BChE.

Amino acid	Non-bonding interactions
HOH706	N9, hydrogen bond-water hydrogen bond; conventional hydrogen bond
HOH706	H34, hydrogen bond-water hydrogen bond; conventional hydrogen bond
HOH825	H58, hydrogen bond-water hydrogen bond; carbon hydrogen bond
HOH825	H59, hydrogen bond-water hydrogen bond; carbon hydrogen bond
Gly116	N9, hydrogen bond-carbon hydrogen bond
Gly116	N9, hydrogen bond-carbon hydrogen bond
Trp82	N1, electrostatic-π-cation
Leu286	C11, hydrophobic-alkyl
Trp231	C11, hydrophobic-π-alkyl
Trp231	C11, hydrophobic-π-alkyl
Phe329	OX-C12, hydrophobic-π-alkyl
Tyr332	OX-C12, hydrophobic-π-alkyl
Phe398	OX-C12, hydrophobic-π-alkyl

Table S25. Interactions between compound QNOH-C_{14} and BChE.

Amino acid	Non-bonding interactions
HOH1031	O10, hydrogen bond-water hydrogen bond; conventional hydrogen bond
HOH884	N9, hydrogen bond-water hydrogen bond; conventional hydrogen bond
Pro285	H27, hydrogen bond-carbon hydrogen bond
Ala328	C11, hydrophobic-alkyl
Phe329	C11, hydrophobic-π-alkyl
Phe329	OX-C14, hydrophobic-π-alkyl
Tyr332	C11, hydrophobic-π-alkyl
His438	OX-C14, hydrophobic-π-alkyl

Table S26. Interactions between compound QNOH-C_{16} and BChE.

Amino acid	Non-bonding interactions
HOH736	O10, hydrogen bond-water hydrogen bond; conventional hydrogen bond
HOH709	N9, hydrogen bond-water hydrogen bond; conventional hydrogen bond

Asp70	H38, hydrogen bond-conventional hydrogen bond
Ile356	C11, hydrophobic-alkyl
Ile356	OX-C16, hydrophobic-alkyl

Table S27. Interactions between compound bisQNOH-C_8 and BChE.

Amino acid	Non-bonding interactions
Glu197	N28, electrostatic-attractive charge
HOH786	N18, hydrogen bond-water hydrogen bond; conventional hydrogen bond
HOH910	O10, hydrogen bond-water hydrogen bond; conventional hydrogen bond
Asp70	H40, hydrogen bond-conventional hydrogen bond
Glu197	H52, hydrogen bond-conventional hydrogen bond
Pro285	H29, hydrogen bond-carbon hydrogen bond
Pro285	H32, hydrogen bond-carbon hydrogen bond
Ser287	H53, hydrogen bond-carbon hydrogen bond
Pro285	H54, hydrogen bond-carbon hydrogen bond
Leu286	OX-BIS-C8, hydrophobic-alkyl

Table S28. Interactions between compound bisQNOH-C_{10} and BChE.

Amino acid	Non-bonding interactions
HOH736	O19, hydrogen bond-water hydrogen bond; conventional hydrogen bond
HOH1031	H41, hydrogen bond-water hydrogen bond; carbon hydrogen bond
Asp70	H54, hydrogen bond-conventional hydrogen bond
Pro285	H34, hydrogen bond-carbon hydrogen bond
Ser287	H40, hydrogen bond-carbon hydrogen bond
Leu286	OX-BIS-C10, hydrophobic-alkyl
Trp231	OX-BIS-C10, hydrophobic-π-alkyl

The cytotoxicity of the selected *N*-alkyl quaternary quinuclidines

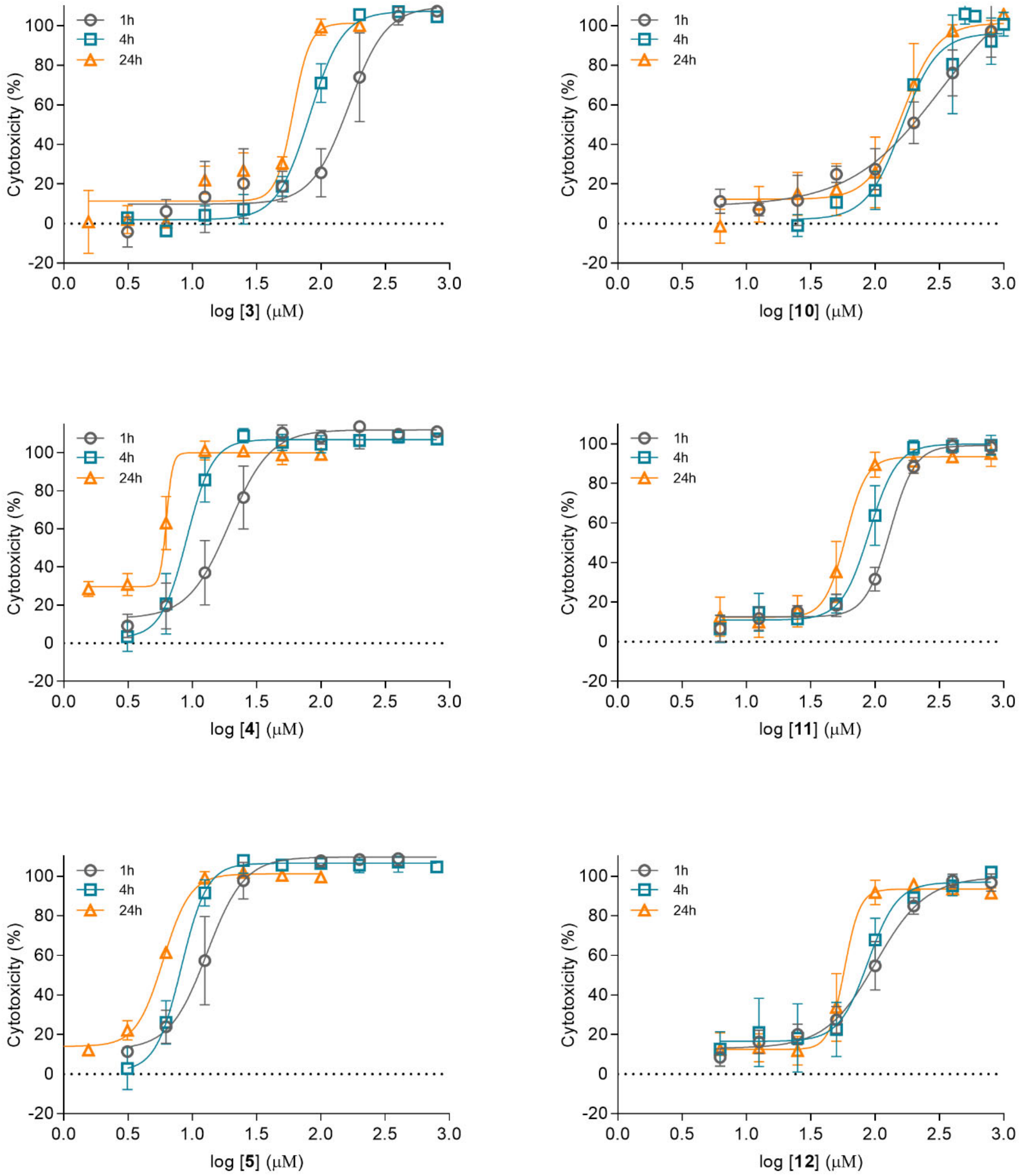

Figure S13. Time and dose-dependent cytotoxicity of selected *N*-alkyl quaternary quinuclidines on SH-SY5Y cells after 1-, 4- and 24-h treatment. Experimental data was presented as a mean of at least three experiments.

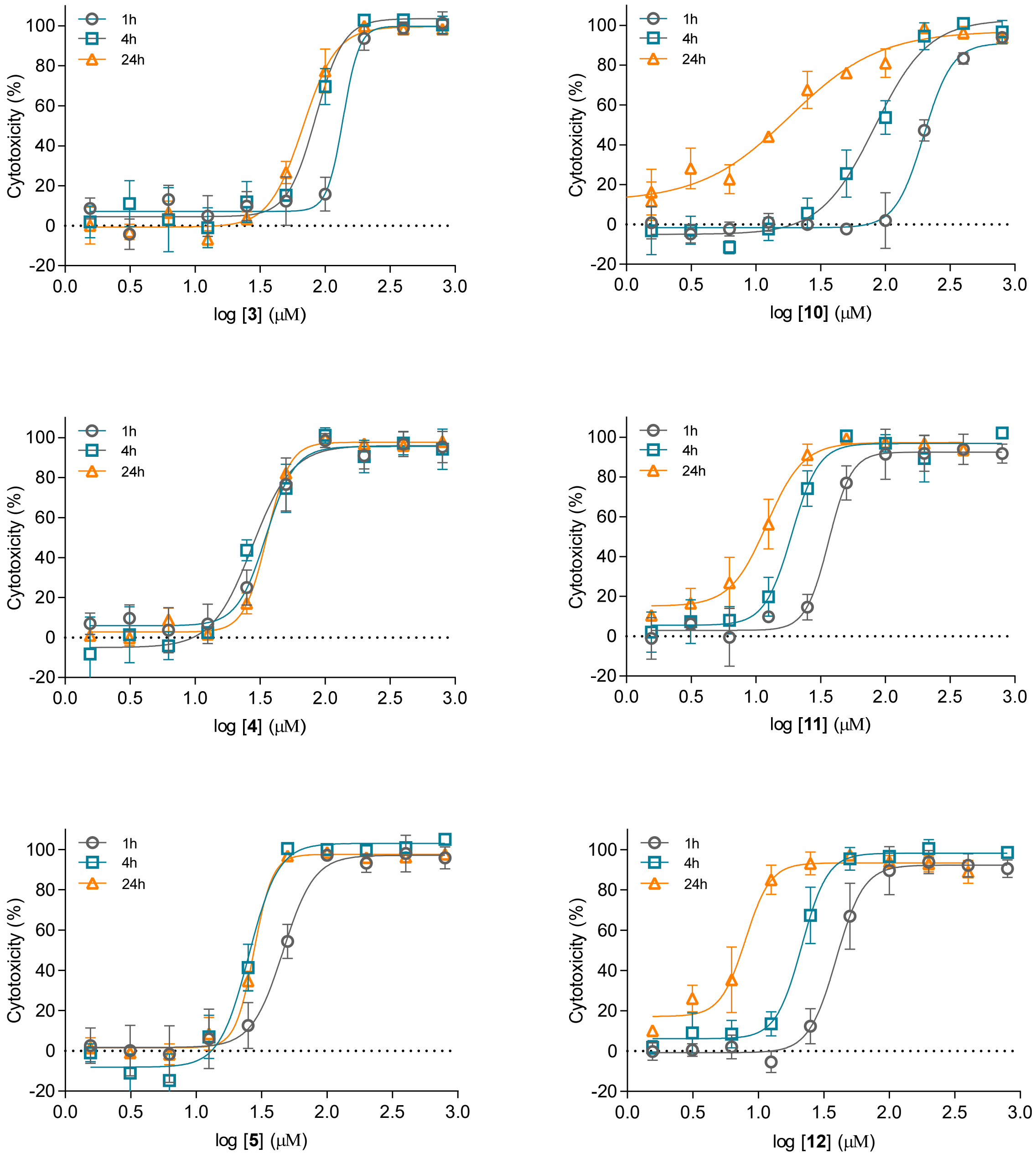

Figure S14. Time and dose-dependent cytotoxicity of selected *N*-alkyl quaternary quinuclidines on HepG2 cells after 1-, 4- and 24-h treatment. Experimental data was presented as a mean of at least three experiments.

Pharmacophore modeling and *in silico* prediction of targets

The pharmacophore models were generated using Common feature pharmacophore generation protocol implemented in Discovery Studio Client. Common feature pharmacophore generation protocol uses HipHop to generate common feature pharmacophores among a set of active ligands. The algorithm can also optionally use information from inactive ligands to place excluded volume features. Experimentally determined IC_{50} values for HepG2 cell line were used as a measure of ligand activity, maximum number of conformations of input ligands to be generated was set to 255 within 20.0 (kcal/mol) relative energy threshold.

In the case of alcohols minimum spacing between feature points was set to 0.5 Å. Pharmacophore features to be used in pharmacophore generation were: HB_ACCEPTOR, HB_DONOR, HYDROPHOBIC_aromatic, HYDROPHOBIC_aliphatic, POS_IONIZABLE, NEG_IONIZABLE, POS_CHARGE, RING_AROMATIC, and iAmide. Number of active molecules in the input set that do not have to map to all features in a generated pharmacophore was set to 8, while the number of molecules in the input set that are allowed not to map to any one feature of a generated pharmacophore was set to 1 and number of molecules input set that do not have to map to more than one feature per generated pharmacophore was set to 8. Separate enantiomers were grouped into two groups, each run individually with Preprocessing parameter left blank and Stereoisomers As Different Molecules parameter set to False.

In the case of oximes, minimum spacing between feature points was set to 2.0 Å. Pharmacophore features to be used in pharmacophore generation were: HB_ACCEPTOR, HB_DONOR, HYDROPHOBIC_aliphatic, POS_IONIZABLE, POS_CHARGE. Number of active molecules in the input set that do not have to map to all features in a generated pharmacophore was set to 4, while the number of molecules in the input set that are allowed not to map to any one feature of a generated pharmacophore was set to 1 and number of molecules input set that do not have to map to more than one feature per generated pharmacophore was set to 4. Preprocessing parameter was set to ClearStereo and ClearUnknownStereo to set all atoms and bonds to NoStereo and/or set all atoms and bonds that are marked UnknownStereo to NoStereo, while Stereoisomers As Different Molecules parameter was set to False.

Finally, among generated pharmacophore models, the representative model was chosen based on greatest sum of FitValues – a measure of how well the ligand fits the pharmacophore – for active compounds.

Search 3D Database protocol using Catalyst was used to identify ligands in a database (DruglikeDiverse, MiniMaybridge, Sample, and scPDB) that map to a pharmacophore and align the returned ligands to the query. The returned ligands were ranked in terms of FitValue. Minimum distance between features of mapping ligands was set to 0.5 Å.

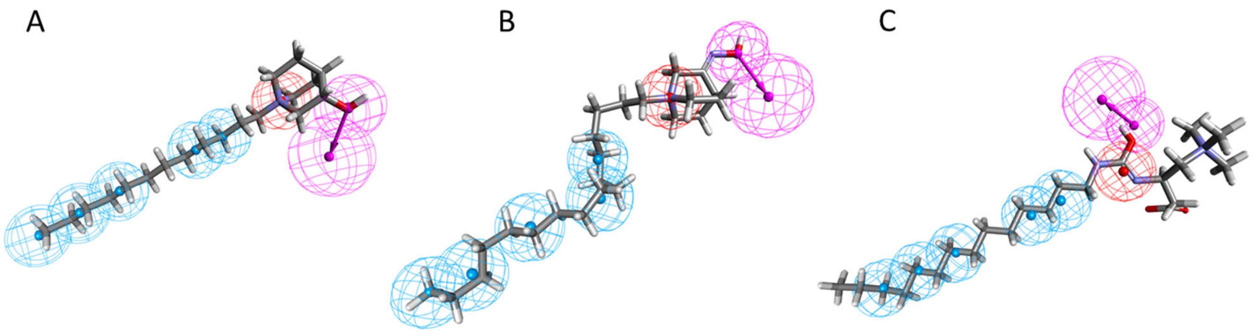

Figure S15. (A) compound **3** (QOH-C_{12}) mapped onto the pharmacophore model for quinuclidine derivates, (B) compound **12** (QNOH-C_{16}) mapped the pharmacophore model for quinuclidine derivates and (C) 3-(tetradecylcarbamoylamino)-4-(trimethylazaniumyl)butanoate mapped onto the pharmacophore model for quinuclidine derivates. Red spheres represent positive ionisable features; magenta spheres represent hydrogen bond donor features; blue spheres represent hydrophobic features.
